# Metabolomic signatures suggest altered bile acid and energy metabolism in *CRB1*- retinopathies

**DOI:** 10.1007/s11306-026-02415-7

**Published:** 2026-04-17

**Authors:** Ana Catalina Rodriguez-Martinez, Neelima Nair, Jane Skinner, Ailsa A. Welch, Samantha Malka, Mariya Moosajee

**Affiliations:** 1 UCL Institute of Ophthalmology, 11-43 Bath Street, London, EC1V 9EL UK; 2 Moorfields Eye Hospital NHS Foundation Trust, London, EC1V 2PD UK; 3 The Francis Crick Institute, London, NW1 1AT UK; 4 Department of Public Health & Primary Care, Norwich Medical School, Norfolk, UK

**Keywords:** CRB1, Crumbs homolog 1, Metabolomics, CRB1-retinopathies, Inherited retinal dystrophies

## Abstract

**Introduction:**

The human *CRB1* gene encodes the CRB1 protein, primarily expressed in retinal Muller cells and photoreceptors, where it regulates apical-basal polarity and cellular signalling through its role in adherence junctions and maintaining the outer limiting membrane barrier. Dysfunction of CRB1 results in a range of retinal phenotypes with few systemic implications reported. It has been suggested that disrupted Crb1 expression in the gastrointestinal epithelium of rd8 mouse models (*Crb1* ^−/−^) results in barrier dysfunction permitting translocation of bacteria to the retina.

**Objective:**

Whole metabolomic analysis in patients can provide further insights into disease pathophysiology and aid the identification of potential systemic biomarkers.

**Methods:**

Blood plasma from 25 molecularly confirmed *CRB1*-retinopathy patients from Moorfields Eye Hospital with 25 age- and gender-matched healthy controls underwent ultra-performance liquid chromatography-mass spectrometry (UPLC-MS). MetaboLync pathway analysis identified affected metabolic pathways.

**Results:**

Of 872 compounds, 244 were significantly altered in *CRB1* patients. Key findings included disrupted bile acid metabolism, with elevated primary and secondary bile acids alongside increased gut microbial phenylalanine pathway metabolites, indicative of altered gut microbiome-related metabolic activity and altered enterohepatic circulation. However, sucrose and butyrate levels remained unchanged amongst groups, suggesting absence of metabolomic evidence for severe intestinal barrier dysfunction. Reductions in antioxidants and neuroprotective agents were found alongside energy metabolism dysregulation.

**Conclusion:**

These findings reveal metabolic dysregulation in *CRB1*-retinopathy, including altered gut microbiome-related metabolic activity, and no strong metabolomic evidence of severe intestinal barrier disruption. The reductions in antioxidants, energy pathways and neuroprotective agents highlight potential therapeutic targets to delay disease progression. Further investigation into gut microbiome composition and intestinal permeability in humans with *CRB1* retinopathies is warranted.

**Supplementary Information:**

The online version contains supplementary material available at 10.1007/s11306-026-02415-7.

## Introduction

The human *CRB1* gene (OMIM #604210), located on chromosome 1q31.3, spans over 210 kb and comprises 12 exons that encode three isoforms: CRB1-A, B, and C (Boon et al. 2020). CRB1 is part of a three-member family of CRB proteins, alongside CRB2 and CRB3; the latter encoding two isoforms, CRB3A and CRB3B. The canonical *CRB1-A* transcript contains 12 exons and is translated into a 1406 amino acid protein (Boon et al. 2020). Its structure includes 19 epidermal growth factor (EGF)-like repeat domains and three laminin-globular-like domains in its extracellular N-terminus, along with a brief FERM/PDZ binding motif in the intracellular C-terminal domain (Boon et al. 2023). It is primarily expressed in Muller glia cells (MGCs) (Mairot et al. 2021). CRB1-B, comprising 1003 amino acids, shares structural similarity with CRB1-A in the extracellular domains but possesses distinctive 5′ and 3′ domains and it is predominantly expressed in photoreceptors (Boon et al. 2020, Mairot et al. 2021, Ray et al. 2020). CRB1-C, comprising 754 amino acids, lacks the transmembrane and intracellular domains, and its function remains uncertain (Alves et al. 2013, Chen et al. 2019).

CRB1 acts as a vital regulator of cellular processes, including apical-basal polarity, outer limiting membrane (OLM) integrity, cell-cell adhesion, and cellular signaling pathways (Boon et al. 2020). It is crucial for retinal development and its long-term integrity, particularly in maintaining zonula adherence junctions at the OLM (Boon et al. 2020, Stehle 2024). Within the adherence junctions, CRB1 is found in complex with PALS1, also known as Membrane Protein Palmitoylated 5 or MPP5, PALS1-associated tight junction protein (PATJ) or the Multi-PDZ Domain Protein 1 (MUPP1) (Shamsnajafabadi et al. 2024, Alves et al. 2014). Together, these proteins regulate signaling pathways, impacting cell proliferation and cell fate, and are critical for the formation of epithelial adherence junctions (Owen et al. 2023). Disruption of the CRB1 complex impairs the polarity and adhesion of the developing retinal epithelium, causing cells to detach from the apical lamina and undergo ectopic mitoses, thereby disturbing the organized, spatiotemporal aspects of retinogenesis (Owen et al. 2023). This disruption results in retinal degeneration and varying degrees of retinal dysfunction, manifesting as a range of retinopathies (Alves et al. 2014) such as Leber congenital amaurosis (OMIM #613935, LCA8), autosomal recessive retinitis pigmentosa (OMIM #600105, RP12), macular dystrophies (MD) and cone-rod dystrophies (CORD) (Rodriguez-Martinez 2023). Notably, patients typically do not exhibit any systemic manifestations into adulthood.

Although CRB members are primarily expressed in the retina (Boon et al. 2020), they are also expressed in extraocular tissues. CRB2 expression is found in tissues such as in kidney podocytes, in the subventricular zone of the brain and in the spinal cord (Boon et al. 2020). While CRB3 is the most widely expressed isoform in epithelial tissues, it is markedly upregulated in the mesenchyme-to-epithelium transition and downregulated in the epithelium-to-mesenchyme transition (Lemmers et al. 2004). A Crb3 KO mouse demonstrated extensive defects in epithelial morphogenesis, dying shortly after birth with cystic kidneys and proteinaceous debris throughout the lungs (Whiteman et al. 2014). While the human protein atlas indicates that CRB1 is expressed in various brain areas, including the cerebral cortex, amygdala, and hippocampus and minimally in other tissues like the ovaries and testes (Boon, et al., 2020, Xiao 2025), systemic expression and function of CRB1 remain under investigation. A recent study using rd8 mouse models (*Crb1* ^−/−^) has revealed Crb1 expression in the gastrointestinal system, specifically on the apical surface of colonic enterocytes, as shown by immunofluorescence staining (Peng et al. 2024). In rd8 mice, this deficiency led to diffuse F-actin (phalloidin) distribution at junctional sites, indicating compromised junction integrity. Transmission electron microscopy further showed disrupted adherens junctions, impairing both the colonic epithelial and outer retinal barriers, referred to as the “leaky gut” and “leaky retina.” The authors stated that this barrier dysfunction allowed translocation of gut flora from the lower gastrointestinal tract to the retina, which contributed to bacteria-dependent retinal degeneration (Peng et al. 2024). However, the implications of CRB1 expression in the human gastrointestinal system remain unclear, and this suggestion of retinal pathophysiology is subject to debate. Furthermore, Moekotte et al. reported elevated levels of IL-17 and IL-23, key Th17 cytokines linked to autoimmune uveitis, along with increased CD4 and CD8 T cells in patients with *CRB1*-retinopathy, suggesting inflammatory pathway involvement in disease pathogenesis (Moekotte et al. 2023). Despite growing evidence of systemic associations in *CRB1*-retinopathy, most research on *CRB1* gene mutations in both humans and mice have primarily focused on the retina. This leaves a significant gap in comprehensive studies exploring the broader systemic effects of *CRB1* gene mutations.

To explore systemic effects of retinal diseases, several studies have analysed blood plasma metabolites. In choroideremia (CHM) patients, deficiency in Rab escort protein 1 (REP1) was found to disrupt lipid metabolism and increase oxidative stress, providing insights into systemic derangements and suggesting potential therapeutic targets (Cunha 2021). In age-related macular degeneration (AMD), metabolomic findings revealed eye-related pathogenic mechanisms such as increased oxidative stress, disrupted lipid and amino acid metabolism, and decreased acylcarnitines, key for energy homeostasis and retinal pigment epithelium (RPE) function (Brown 2018). Given the complex genetic, epigenetic and environmental interactions in *CRB1*-retinopathy that complicate understanding disease mechanisms, metabolomic analysis may identify metabolic changes offering system-level insights into the *CRB1* pathophysiology. This study aims to identify systemic biomarkers for *CRB1*-related retinopathy through whole blood metabolomic analysis, potentially offering new insights into disease mechanism, diagnostic tools and surrogate outcome measures in clinical trials.

## Methods

### Clinical evaluation

Twenty-five unrelated patients with clinically and molecularly confirmed autosomal recessive *CRB1*-retinopathies from Moorfields Eye Hospital NHS Foundation Trust (London, UK), along with 25 age- and gender-matched controls, were included in the study. Potential subjects were identified from the prospectively consented Moorfields Eye Hospital Inherited Eye Disease Database for structure/function of genetic diseases (Research Ethics Number: 12/LO/0141) and all procedures adhered to the tenets of the Declaration of Helsinki. Comprehensive ophthalmologic examinations were conducted as part of routine care. Full details of demographic characteristics of this cohort are reported in Supplementary Table 1. Patients with a history of diabetes, hypercholesterolemia, or those taking statins or other medications were excluded from the study.

### Assessment of dietary intake

All *CRB1* patients and controls completed a Food Frequency Questionnaire (FFQ) concerning their consumption frequency of various foods and beverages over the past 12 months. This validated FFQ included 147 food items, covering multiple dimensions such as fruits, vegetables, meats, dairy products, and beverages. Participants were asked to select their usual consumption frequency for each food item from nine frequency categories, ranging from “never or less than once a month” to "six or more times a day" (Welch, et al., 2005). Additionally, if patients frequently consumed foods outside the 147 listed items or supplements such as vitamins, fish oil, etc., they were also required to record these. Individuals were excluded if more than 10 items were left unanswered and this did not apply to any participants. Nutrient calculations were performed using the UK nutritional database (Bingham et al. 1997). See supplemental Table 2.

### Sample collection

Blood samples were collected from non-fasting *CRB1* patients (*n* = 25) and age- and gender-matched healthy individuals (*n* = 25). Blood plasma was extracted by centrifuging whole blood at 600 g for 15 min at room temperature. The extracted plasma samples were aliquoted and stored at − 80 °C. Samples that had not been previously thawed were shipped on dry ice to Metabolon Inc.

### Metabolomics analysis

Blood plasma metabolite extractions for ultra-performance liquid chromatography-mass spectrometry (UPLC-MS) were completed by Metabolon Inc., according to the protocol described in Supplemental Material. To visualize and analyse small molecules within relevant networks of metabolic pathways, the detected metabolites in *CRB1* patients and control study groups were subjected to MetaboLync pathway analysis (MPA) software (www.portal.metabolon.com).

### Statistics

An estimate of the false discovery rate (FDR) (q value) was calculated considering multiple comparisons that normally occur in metabolomic-based studies, and a threshold of *q* ≤ 0.10 was used to correct for false discovery of statistically significant compounds. FC was determined by dividing the relative abundance of each metabolite in the *CRB1* patient’s blood plasma by the relative abundance of the metabolite in the blood plasma of healthy control individuals. FC values with *p* ≤ 0.05 and q ≤ 0.10 were considered statistically significant, while FC values with 0.05 < *p* < 0.10 were considered as trending toward significance Welch’s two-sample t-test was used.

## Results

### Patient description

Twenty-five molecularly confirmed *CRB1*-retinopathy patients were included, with a mean age (± SD) of 25.0 years (± 14.6) (range 6–53 years) at the time of blood collection. Among the 25 patients, 11 were male and 14 were female. Ten patients presented with the LCA/EOSRD phenotype, nine with MD, four with CORD, and one with RP. Twenty-five controls were included with a mean age of 36 years (± 16.4) (range 12–69 years), 12 were male and 13 were female. Detailed clinical and genetic information is included in Supplemental Table 1. Analysis of the FFQ results revealed no significant dietary differences between disease and control groups with regards to average consumption of food and beverages over the past 12 months except for caffeine intake, which was significantly less (*p* < 0.01) in the *CRB1* group (75.0 ± 75 mg) compared to the control group (174.8 ± 134.2 mg). (see Table 1)

**Table 1:** Summary of subject demographics, genetic results, and clinical characteristics of the 25 patients with biallelic pathogenic variants in *CRB1*

Family number	Subject	Gender	Ethnicity	Age	Phenotype	Zygosity	Variant 1 cDNA Variant 1 protein	Variant 2 cDNA Variant 2 protein
45,590	01	F	Black	29	MD	Homozygous	c.2506 C > A p.Pro836Thr	
46,120	02	F	White	17	EOSRD/LCA	Heterozygous	c.455G > A p.Cys152Tyr	c.3014 A > T p.Asp1005Val
35,083	03	M	White	39	MD	Heterozygous	c.498_506del p.Ile167_Gly169del	c.4142 C > G p.Pro1381Arg
43,560	04	F	White	48	MD	Heterozygous	c.498_506del p.Ile167_Gly169del	c.1696G > T p.Glu556Ter
38,236	05	M	White	47	MD	Heterozygous	c.498_506del p.Ile167_Gly169del	c.584G > T p.Cys195Phe
16,285	06	M	White	26	EOSRD/LCA	Homozygous	c.2843G > A p.Cys948Tyr	
Z889804	07	M	Asian	13	CORD	Heterozygous	c.498_506del p.Ile167_Gly169del	c.4005 + 1G > A N/A
32,309	08	M	White	15	MD	Heterozygous	c.498_506del p.Ile167_Gly169del	c.1576 C > T p.Arg525*
42,270	09	M	White	52	MD	Heterozygous	c.498_506del p.Ile167_Gly169del	c.2401 A > T p.Lys801*
31,953	10	F	White	16	EOSRD/LCA	Heterozygous	c.2548G > A p.Gly850Ser	c.4006–10 A > G N/A
37,161	11	M	White	17	MD	Heterozygous	c.498_506del p.Ile167_Gly169del	c.2308G > T p.Gly770Cys
46,830	12	M	White	10	EOSRD/LCA	Heterozygous	c.2843G > A p.Cys948Tyr	c.1712 A > C p.Glu571Ala
44,092	13	F	White	11	MD	Heterozygous	c.498_506del p.Ile167_Gly169del	c.2843G > A p.Cys948Tyr
35,283	14	M	White	11	EOSRD/LCA	Homozygous	c.2843G > A p.Cys948Tyr	
29,882	15	F	White	16	EOSRD/LCA	Homozygous	c.14559T > C p.Ser487Pro	
32,038	16	M	White	40	CORD	Heterozygous	c.498_506del p.Ile167_Gly169del	c.1431delG p.Ser478Profs*24
33,707	17	M	White	29	MD	Heterozygous	c.498_506del p.Ile167_Gly169del	c.3827_3828del p.Glu1276Valfs*4
47,941	18	M	White	18	EOSRD/LCA	Heterozygous	c.2291G > A p.Arg764His	
21,819	19	M	White	33	CORD	Homozygous	c.470G > C p.C157Sp.Cys157Ser	c.2506 C > A p.Pro836Thr
35,229	20	F	White	34	MD	Heterozygous	c.498_506del p.Ile167_Gly169del	c.2290 C > T p.Arg764Cys
27,040	21	F	White	5	EOSRD/LCA	Heterozygous	c.2401 A > T p.Lys801*	c.2688T > A p.Cys896*
28,183	22	F	White	5	EOSRD/LCA	Heterozygous	c.1339dupC p.His447Prof*7	c.2401 A > T p.Lys801*
	23	F	White	41	MD	Heterozygous	c.498_506del p.Ile167_Gly169del	c.3718T > A p.Cys1240Ser
18,067	24	M	White	16	EOSRD/LCA	Heterozygous	c.2401 A > T p.Lys801*	c.2872delAG p.S958fs
24,729	25	M	Asian	13	EOSRD/LCA	Heterozygous	c.2512 A > T p.Lys838*	c.3676G > T p.Gly1226*

### Global metabolite differences between *CRB1* patients and controls

**Fig. 1:**
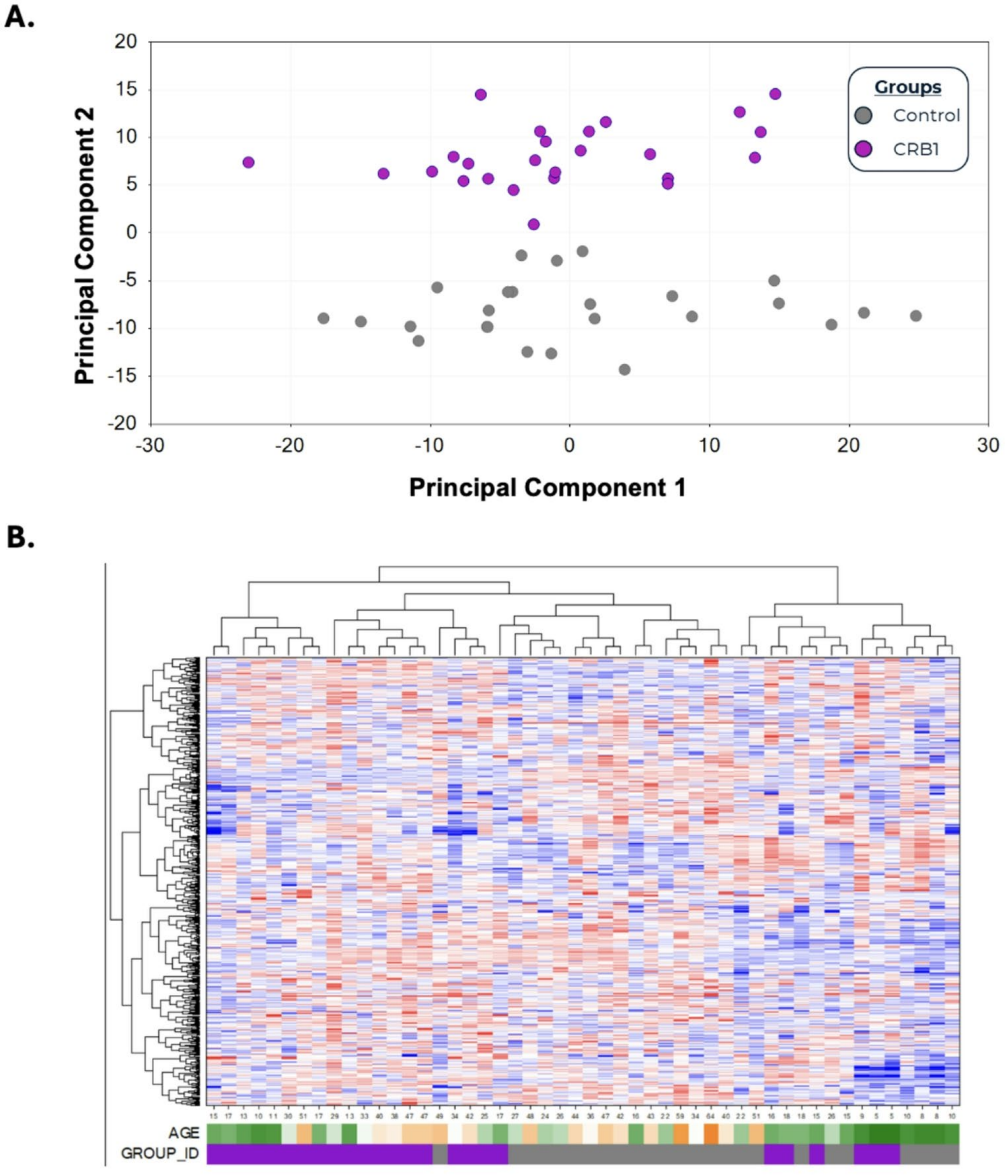
**A** Principal Component Analysis (PCA) of *CRB1* and control samples, samples are represented as purple in the *CRB1* group and grey in the control group (*n* = 25 each group). PCA and hierarchical clustering demonstrate no overlap between *CRB1* patients and controls, with clear group separation **B**. Cluster analysis of control and *CRB1* samples show clear separation between groups

**Fig. 2:**
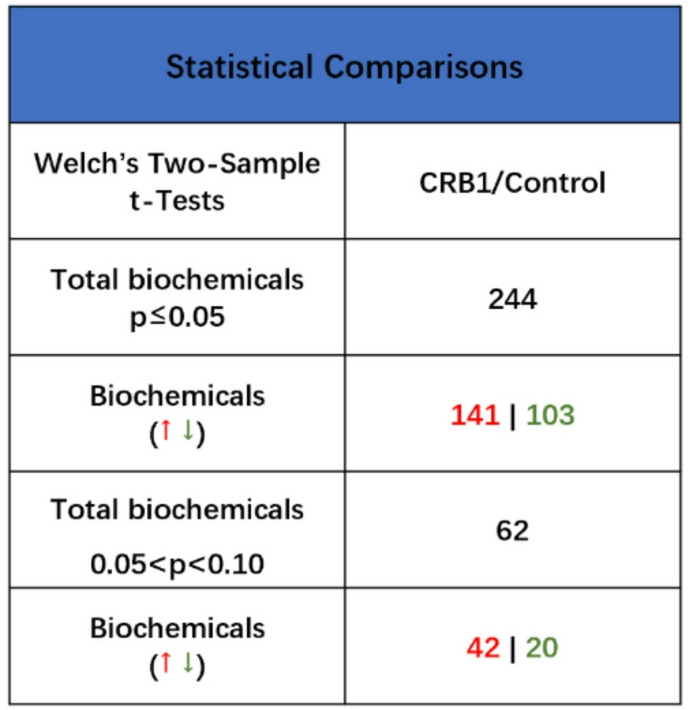
Differential plasma metabolites between ***CRB1*** patients and controls. The number of biochemical compounds in the plasma of *CRB1* patients showing significant and approaching significant changes, as determined by Welch’s two-sample t-test. Red indicates an increase, while green indicates a decrease

Of 872 identified compounds detected in the *CRB1* group plasma and control group, 141 showed significant elevation and 103 showed significant reduction in *CRB1* patients (*p* ≤ 0.05), with an additional 62 compounds approaching significance (0.05 < *p* < 0.10) (42 elevated, 20 reduced) (Fig. 2). Principal Component Analysis (PCA) demonstrated no overlapping grouping with clear distinction between *CRB1* patients and controls (Fig. 1A). Similarly, hierarchical clustering analysis confirmed this trend, showing that control and *CRB1* patients tend to cluster apart, suggesting that there are differences between the metabolomes of these two cohorts (Fig. 1B). No supervised classification analyses were performed due to the exploratory nature of the dataset and potential technical variability introduced during data harmonisation.

#### *CRB1* patients display altered enterohepatic metabolism

**Fig. 3:**
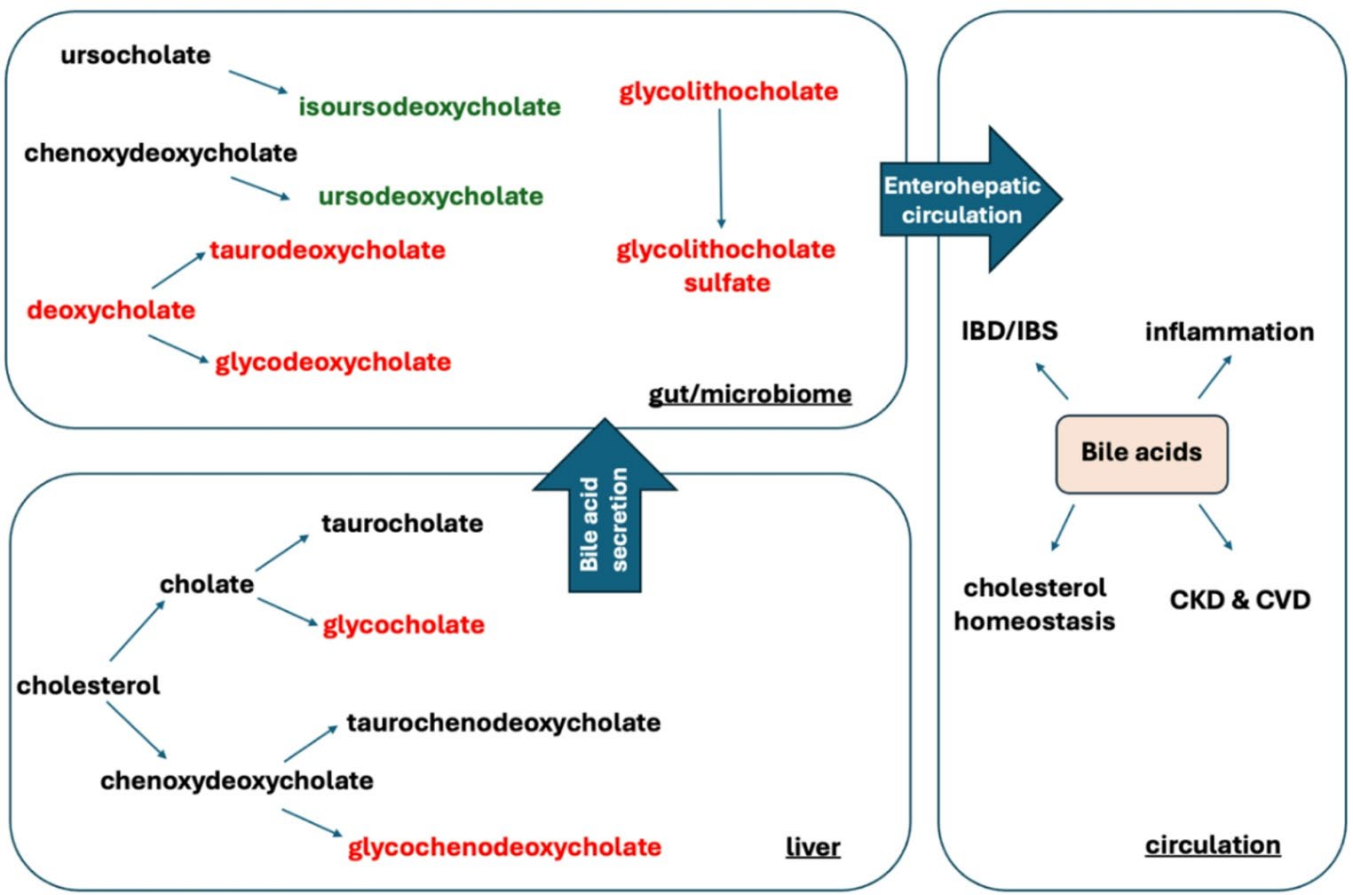
Pathway diagram of bile acid metabolism and enterohepatic circulation. Metabolites with significant increases (*p* ≤ 0.05) are colour coded in red, while those with significant decreases (*p* ≤ 0.05) are colour coded in green. While individual fold changes are modest, the coherence across related metabolites suggests pathway-level alterations. *CKD Chronic kidney disease, *CVD *Chronic cardiovascular disease, *IBD* Inflammatory bowel disease, *IBS* Irritable bowel syndrome

**Fig. 4:**
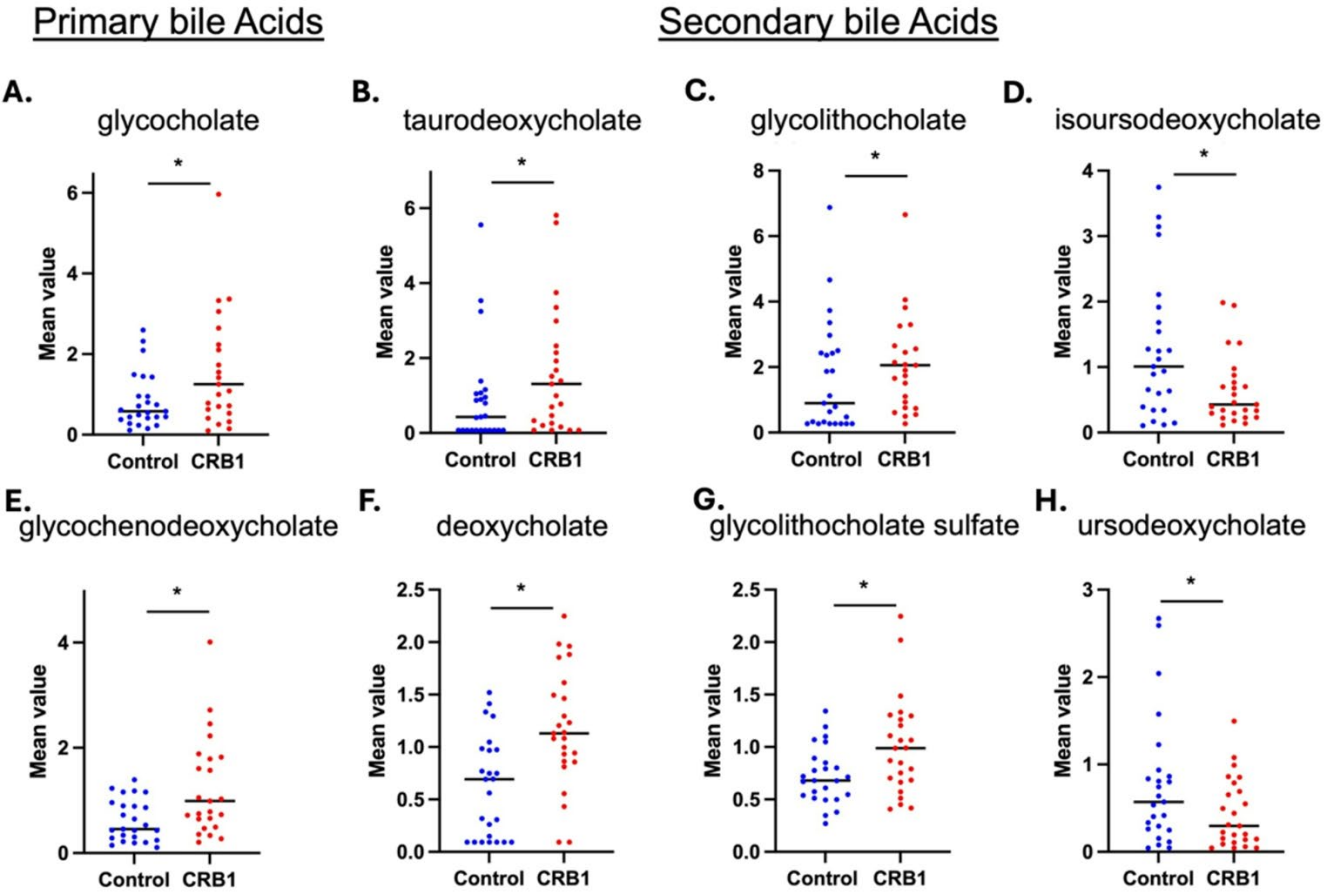
*CRB1* patients show an increase in several primary and secondary bile acids. Box plots display the mean ± SD levels in *CRB1* patients (red) compared to control samples (blue) (*n* = 25). Primary bile acids such as (**A**) glycocholate (FC 2.71, *p* ≤ 0.05) and **E**. glycochenodeoxycholate (FC 2.38, *p* ≤ 0.05) were found to be statistically significantly elevated. Similarly, increased levels in secondary bile acids like (**B**) taurodeoxycholate (FC 2.22, *p* ≤ 0.05) (**C**) glycolithocholate (FC 2.01, *p* ≤ 0.05), **F**. deoxycholate (FC 1.89, *p* ≤ 0.05) and **G**. glycolithocholate sulfate (FC 1.77, *p* ≤ 0.05) were observed. **D** isoursodeoxycholate (FC 0.5, *p* ≤ 0.05), **H**. ursodeoxycholate (FC 0.57, 0.05 < *p* < 0.10) were found to be statistically significant reduced in *CRB1* patients

Bile acids are the final products of cholesterol metabolism (Su et al. 2023). They are synthesized in the liver as primary bile acids such as cholic acid, chenodeoxycholic acid and their conjugated forms with glycine, taurine, or other amino acids. They are then secreted into the small intestine, where these conjugated bile acids are modified by various bacterial enzymes, resulting in the formation of secondary bile acids (Su et al. 2023). The primary and secondary bile acid metabolism pathways in the *CRB1* group showed fluctuations (Fig. 3). Primary bile acids glycocholate (FC 2.71, *p* ≤ 0.05), glycochenodeoxycholate (FC 2.38, *p* ≤ 0.05), and glycochenodeoxycholate glucuronide (FC 1.44, 0.05 < *p* < 0.10) all exhibited a significant increase. The increase in glycocholate and glycochenodeoxycholate reflected an upregulation of bile acid conjugation with glycine, with the latter showing a significant increase (FC 1.17, *p* ≤ 0.05). (Fig. 4)

The gut microbiome engages in two main reactions with primary bile acids: redox reactions and deconjugation reactions. Redox reactions primarily involve the transformation of primary bile acids into secondary bile acids through intestinal processes. Secondary bile acids produced by redox reactions, such as deoxycholate (FC 1.89, *p* ≤ 0.05), glycodeoxycholate (FC 2.36, 0.05 < *p* < 0.10), and taurodeoxycholate (FC 2.22, *p* ≤ 0.05) were increased. Conversely, deconjugation reactions performed by the gut microbiome mainly involve the hydrolysis of conjugated bile acids by hydrolases, releasing unconjugated forms of bile acids. Bile acids produced by deconjugation reactions, such as glycolithocholate (FC 2.01, *p* ≤ 0.05), glycolithocholate sulfate (FC 1.77, *p* ≤ 0.05), glycohyocholate (FC 1.75, 0.05 < *p* < 0.10), glycocholenate sulfate (FC 1.38, *p* < 0.05), taurocholenate sulfate (FC 1.46, *p* ≤ 0.05), and taurolithocholate 3-sulfate (FC 1.68, *p* ≤ 0.05), were all significantly increased. Glycohyocholate which was not significantly increased (FC 1.75, 0.05 < *p* < 0.10) suggested enhanced sulfation of metabolites, an important pathway for bile acid detoxification. Additionally, the decrease in ursodeoxycholate (FC 0.57, 0.05 < *p* < 0.10) and isoursodeoxycholate (FC 0.5, *p* ≤ 0.05) reflected abnormalities in their synthesis or transformation processes. Overall, bile acid metabolism in patients showed significant disruption, which may be attributed to changes in the enterohepatic circulation (bile secretion, uptake from the digestive tract, or liver filtration and circulation) (Fig. 4).

**Fig. 5:**
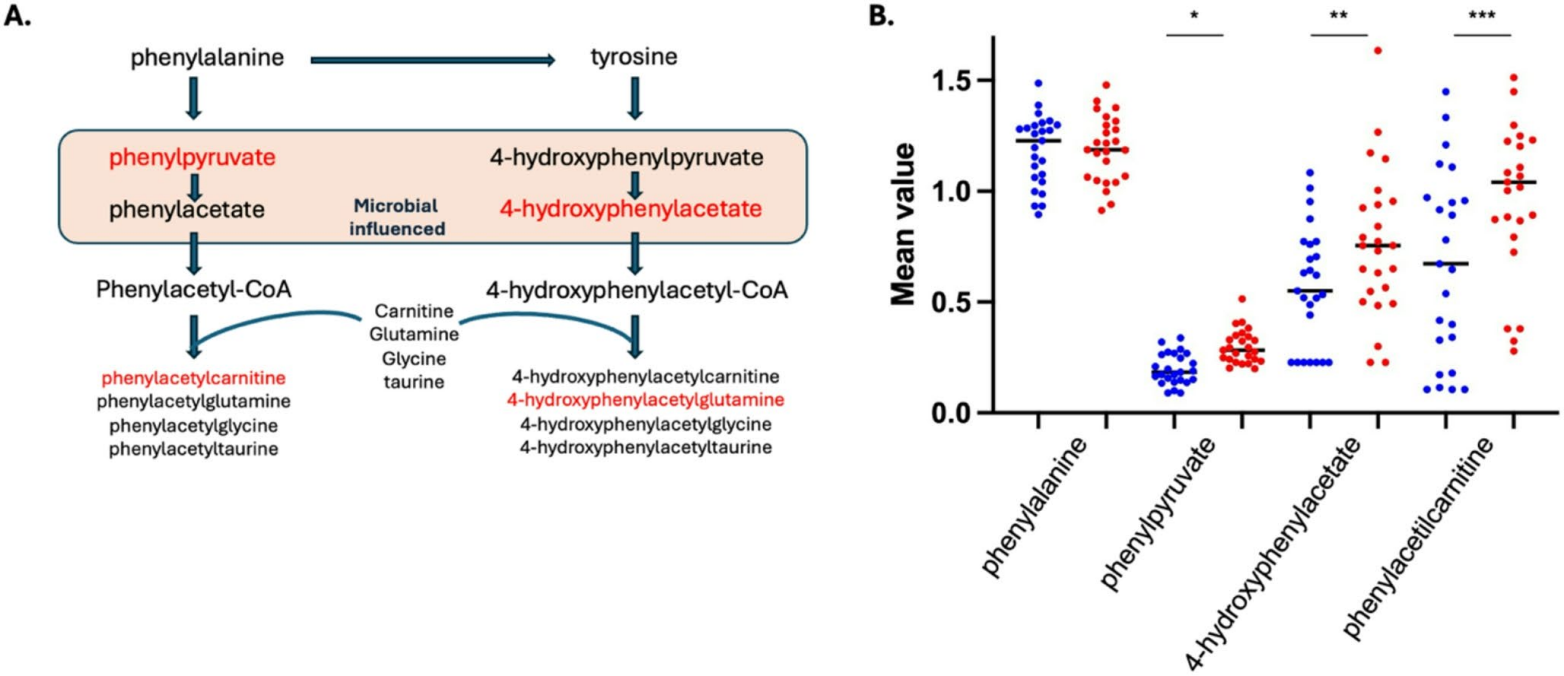
Dysregulation of the phenylalanine pathway was observed in *CRB1* patients. Box plots display the mean ± SD levels in *CRB1* patients (red) compared to control samples (blue) (*n* = 25). **A** Depiction of phenylalanine pathway (**B**) No statistically significant difference between the levels of phenylalanine between both groups, whereas phenylpyruvate, (*FC 1.51, *p* ≤ 0.05), 4-hydroxyphenylacetate (**FC 1.72, *p* ≤ 0.05), and phenylacetylcarnitine (*** FC 1.34 *p* ≤ 0.05) were found significantly elevated in *CRB1* patients

Dysregulation of the phenylalanine pathway was also observed, despite dietary intake of phenylalanine remaining within normal limits for the *CRB1* group compared to controls. In the colon, gut bacteria convert dietary phenylalanine into phenylpyruvate, which is then processed into phenylacetate through oxidative and nonoxidative decarboxylation. In *CRB1* patients, phenylpyruvate (FC 1.51, *p* ≤ 0.05), 4-hydroxyphenylacetate (FC 1.72, *p* ≤ 0.05), phenylacetylcarnitine (*** FC 1.34 *p* ≤ 0.05), and 4-hydroxyphenylacetylglutamine (FC 1.21, *p* ≤ 0.05) were significantly elevated compared to controls, indicating increased microbial activity in the gut from phenylalanine (Fig. 5).

#### *CRB1* patients exhibit evidence of reduction of key antioxidants

**Fig. 6:**
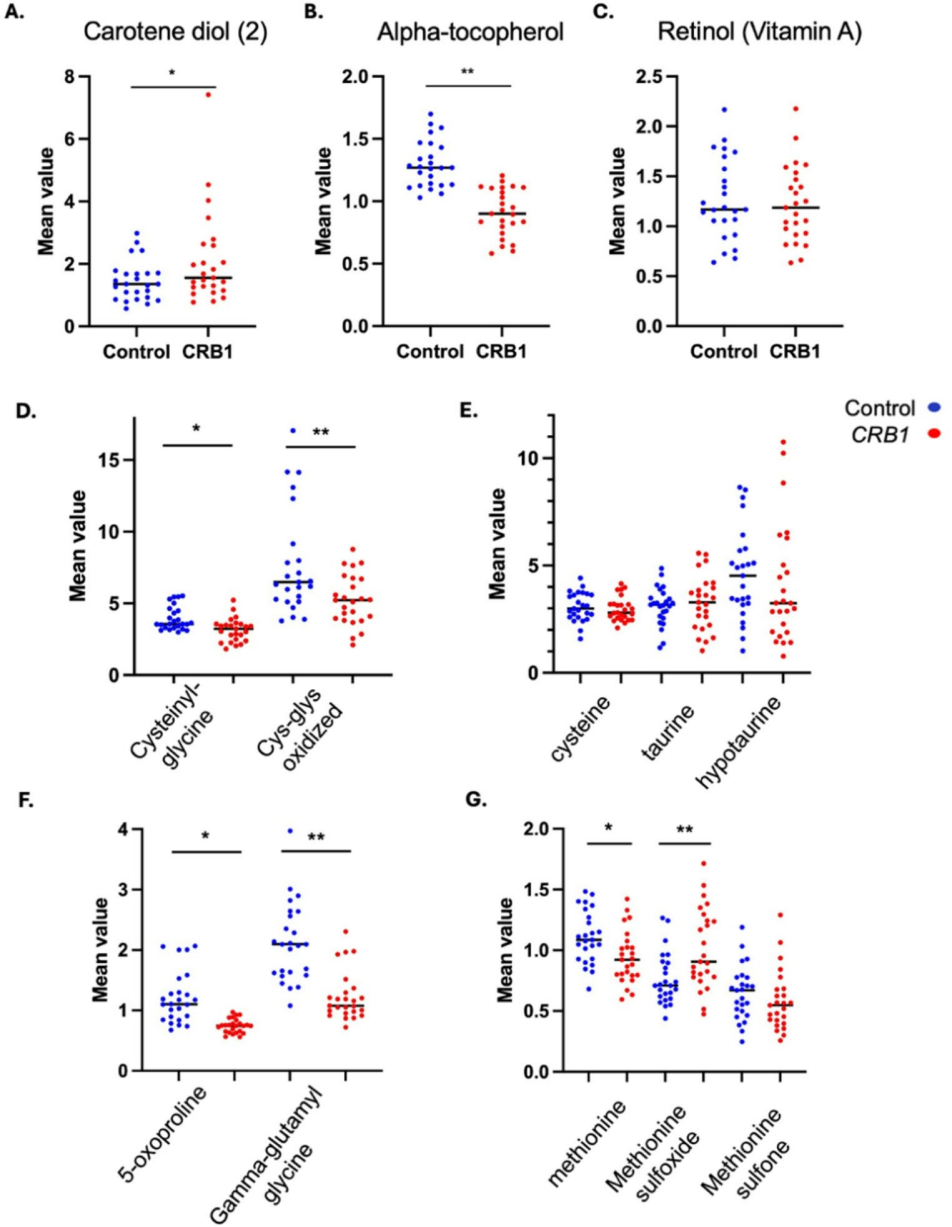
*CRB1* patients exhibit evidence of reduction of a key antioxidant, alpha-tocopherol, and no abnormality in oxidative stress markers. Box plots indicating the mean ± SD levels in *CRB1* patients (red) and control samples (blue) (*n* = 25) of carotene diol (2), alpha-tocopherol and retinol (vitamin A). **A** Carotene diol (2), a vitamin A precursor was found increased (*, FC 1.45, *p* ≤ 0.05). **B** α-tocopherol levels were found reduced in *CRB1* patients (**, FC 0.7, *p* ≤ 0.05). **C** Retinol (vitamin A) levels were similar on both groups. **D** Cysteinylglycine elevated (*FC 0.35, *p* ≤ 0.05) and cys-glys oxidized levels (**FC 0.63, *p* ≤ 0.05) were found statistically significantly lower in *CRB1* patients compared to controls. **E** Whereas, cysteine, taurine and hypotaurine levels were found similar in both groups. **F** 5-oxoproline (*FC 0.61, *p* ≤ 0.05) and gamma-glutamylglycine (**FC 0.58, *p* ≤ 0.05) in *CRB1* patients. **G** Methionine (*FC 0.61, *p* ≤ 0.05), and methionine sulfoxide (*FC 0.61, *p* ≤ 0.05) were found to be significantly lower in *CRB1* patients, while methionine sulfone was found unchanged between groups

A significant difference was observed in both the vitamin A and tocopherol (vitamin E) metabolic pathways (Fig. 6). In the vitamin A pathway, carotene diol (2) levels were elevated in the *CRB1* group (FC 1.45, *p* ≤ 0.05), which is primarily a precursor to vitamin A and functions as an antioxidant. However, changes in vitamin A and other related metabolites were not significant. In the tocopherol pathway, a significant decrease in α-tocopherol levels was observed (FC 0.7, *p* ≤ 0.05), indicating a reduction in this key antioxidant in the patient group. Other metabolites such as gamma-CEHC, gamma-CEHC glucoside, and γ-tocopherol/β-tocopherol were not statistically significant. Other relevant antioxidants such as bilirubin (FC 0.47, *p* ≤ 0.05), ergothioneine (FC 0.30, *p* ≤ 0.05) were also found significantly reduced in the *CRB1* group.

There was no conclusive evidence for significant alterations in redox balance in the *CRB1* group compared to controls (Fig. 6). Specifically, relevant disulfides, including cysteinylglycine disulfide (FC 0.8) and oxidized cys-gly (FC 0.63), were less abundant in the *CRB1* group, suggesting lower oxidative stress (Fu et al. 2019). Additionally, methionine oxidized products, such as methionine (FC 0.85), methionine sulfoxide (FC 1.32), and methionine sulfone (FC 0.9), were also reduced, further supporting the notion of diminished oxidative stress (Suzuki et al. 2016), with a shift in the ratio favouring sulfoxide over sulfone. Furthermore, there is a lack of change in free cysteine (typically the rate limiting component of GSH synthesis), which suggests there is no drive for expansion (i.e. *de novo* synthesis) of the glutathione pool. In addition, taurine metabolism which has been linked to oxidative stress appeared grossly unchanged, this included taurine (FC 1.05) and other relevant taurine products such as cysteine (FC 0.97) and hypotaurine (FC 0.88). Finally, the study observed a downregulation in gamma-glutamyl amino acid (GGAA) metabolism within the *CRB1* group. This was accompanied by a reduction in 5-oxoproline levels, indicating a potential decrease in cystine regeneration through the salvage pathway, which depends on GGAA activity (Ndrepepa and Kastrati 2016).

#### *CRB1* patients exhibit abnormal energetics metabolism

**Fig. 7:**
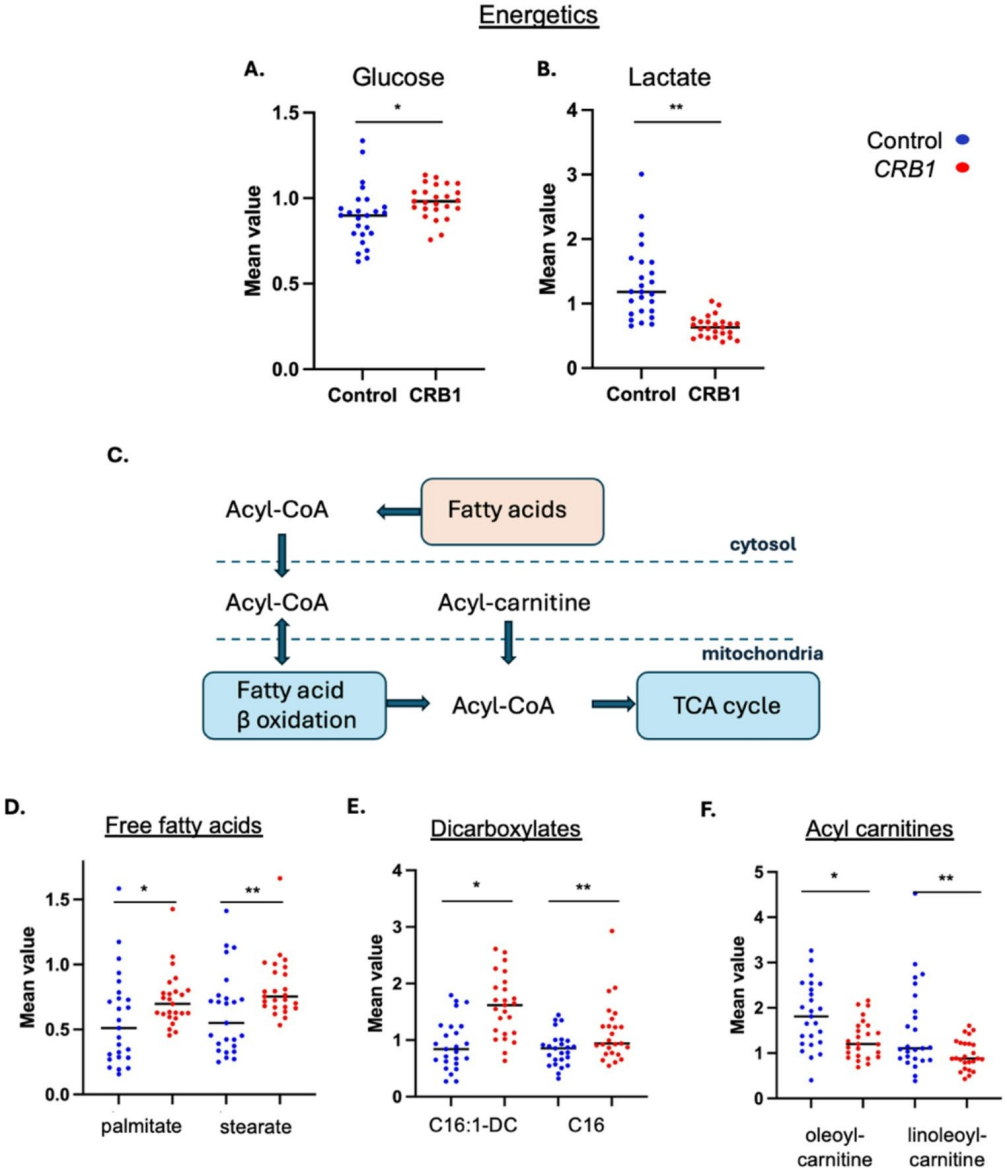
*CRB1* patients exhibit abnormal metabolite levels associated with energetics with abnormal fatty acid oxidation.** A** Glucose level was significantly higher in *CRB1* patients (*FC 1.09, *p* ≤ 0.05). **B** Lactate levels were found statistically significantly lower (**FC 0.49, *p* ≤ 0.05). **C** Schematic representation of fatty acid β-oxidation. **D** Significant increases were observed in palmitate (16:0) (FC 1.27, *p* ≤ 0.05) and stearate (18:0) (FC 1.31, *p* ≤ 0.05) within the *CRB1* group. **E** Similarly, dicarboxylates such as hexadecanedioate (C16-DC) (FC 1.35, *p* ≤ 0.05) and hexadecenoyldioic acid (C16:1-DC) (FC 1.74, *p* ≤ 0.05) were significantly elevated in the *CRB1* group. **F** In contrast, levels of acylcarnitines, including oleoylcarnitine (C18:1) (FC 0.71, *p* ≤ 0.05) and linoleoylcarnitine (C18:2) (FC 0.64, *p* ≤ 0.05), were significantly lower in *CRB1* patients

Despite similar dietary intake of energetics among the *CRB1* patients and controls, glucose levels were significantly increased in the *CRB1* group (FC 1.09, *p* ≤ 0.05), whereas lactate levels were significantly reduced (FC 0.49, *p* ≤ 0.05) (Fig. 7). The significant decrease in lactate levels may be due to its rapid conversion to pyruvate. Pyruvate is quickly utilized in the TCA cycle, converting to acetyl-CoA and entering the TCA cycle for further energy production.

Levels of free fatty acids, primarily released by adipose tissue or from capillary bed lipase activity on lipoproteins, were elevated within the *CRB1* group compared to controls, with significant increases observed in palmitate (16:0) (FC 1.27, *p* ≤ 0.05) and stearate (18:0) (FC 1.31, *p* ≤ 0.05) (Fig. 7). Similarly, the dicarboxylic fatty acid metabolic pathway was increased, with significantly higher levels of hexadecanedioate (C16-DC) (FC 1.35 *p* ≤ 0.05) and hexadecanedioate (C16:1-DC) (FC 1.74 *p* ≤ 0.05). Dicarboxylates are often elevated when lipid mobilization exceeds mitochondrial capacity for lipid uptake (either due to increased mobilization, or reduced uptake). Conversely, levels of acylcarnitines such as oleoylcarnitine (C18:1) (FC 0.71, *p* ≤ 0.05) and linoleoylcarnitine (C18:2) (FC 0.64, *p* ≤ 0.05), both involved in the utilization or transport of unsaturated fatty acids, were statistically significantly lower in *CRB1* patients suggestive of decreased lipid oxidation. Finally, there were no major changes in the pentose phosphate pathway for NADH regeneration and TCA cycle.

#### *CRB1* patients showed no strong metabolomic evidence of severe gut epithelial barrier disruption and no abnormalities in kidney function markers

Sucrose levels showed no significant difference between the *CRB1* group and controls. Under normal conditions, disaccharides like sucrose (molecular weight: 342.3 g/mol) cannot cross an intact intestinal barrier. Instead, it is enzymatically broken down at the microvillus membrane (brush border) of enterocytes in the small intestine into their monomers, glucose and fructose (each with a molecular weight of 180 g/mol). These monomers are then absorbed via specific transporters, such as SGLT1 for glucose and GLUT5 for fructose in the small intestine. If the gut barrier is compromised in the small intestine, intact sucrose could bypass this process and directly enter the bloodstream. Similarly, butyrate levels showed no significant difference between control. Butyrate is a short-chain fatty acid primarily produced in the colon by bacterial fermentation of dietary fibre. Its absorption in the colon occurs primarily through monocarboxylate transporter 1 (MCT1/SLC16A1) and sodium-coupled monocarboxylate transporter 1 (SMCT1/SLC5A8) on the apical membrane of colonocytes (Peng et al. 2007). Normal butyrate levels in *CRB1* patients suggest preserved colonic microbial fermentation and epithelial uptake; however, circulating butyrate concentration are influenced by multiple physiological factors. While this indicates normal transcellular transport, it does not directly confirm tight junction integrity, as butyrate primarily traverses through cell membranes rather than paracellular pathways. However, butyrate is known to enhance barrier function by increasing tight junction proteins (claudin-1, ZO-1), so normal levels indirectly support no strong evidence of severe gut epithelial barrier disruption (Peng et al. 2007).

**Fig. 8:**
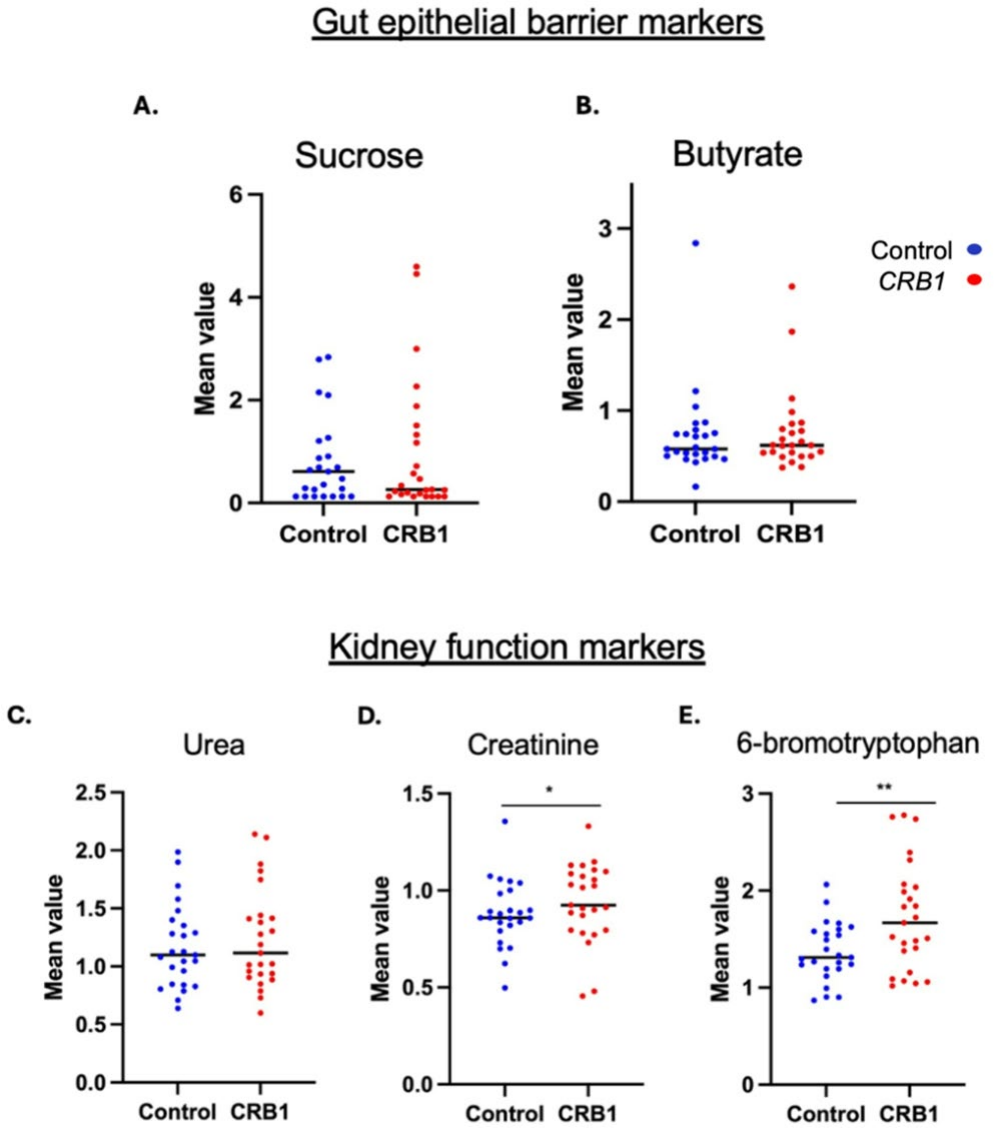
*CRB1* patients did not exhibited strong metabolite evidence of gut barrier disruption or abnormalities in kidney function markers.** A-B**. Plasma levels of sucrose and butyrate did not differ markedly between CRB1 patients and controls (**C**) No statistically significant difference was observed in the mean values of urea levels between *CRB1* patients and controls. **D** Creatinine levels (*FC 0.85, *p* ≤ 0.05) were significantly lower in the *CRB1* group. **E** 6-bromotryptophan was elevated (**FC 1.26, *p* ≤ 0.05) in *CRB1* patients

Since CRB members are expressed in kidney podocytes and epithelial cells, markers of kidney function were explored (Boon et al. 2020). Urea levels were within normal limits (Fig. 8) in *CRB1* patients. Creatinine levels (FC 0.85, *p* ≤ 0.05), were significantly lower in the *CRB1* group, which is another important marker for kidney function. Conversely, 6-bromotryptophan was elevated (FC 1.26, *p* ≤ 0.05). However, studies have shown that higher serum levels of 6-bromotryptophan are linked to a reduced risk of chronic kidney disease (CKD) progression (Sekula et al. 2020, Tin et al. 2018). Lower creatinine levels observed in *CRB1* patients may partially reflect the younger mean age of this cohort, as creatinine correlates with age and muscle mass.

## Discussion

This comprehensive metabolomic analysis of patients with *CRB1*-retinopathies offers new insights into systemic disruptions, revealing significant alterations in multiple metabolic pathways beyond the retina. Key findings include disrupted bile acid metabolism with elevated levels of both primary and secondary bile acids, reduced antioxidants and neuroprotective agents, and abnormalities in energy metabolism.

Bile acids, produced in the liver from cholesterol, are released into the small intestine, where they can be modified by the gut microbiome, leading to the formation of secondary bile acids as they pass through the digestive system (Urdaneta and Casadesús 2017). Secondary bile acids were significantly increased in *CRB1* patients compared to controls. Notably, glycolithocholate and deoxycholate−two of the increased bile acids− are known to modulate innate immunity and T cell activation by activating bile acid receptors. Deoxycholate activates the Sphingosine-1-Phosphate Receptor 2 (S1PR2) which enhances the expression of adhesion molecules like ICAM-1 and VCAM-1, facilitating lymphocyte migration to the intestine and exacerbating inflammation. In addition, deoxycholate can activate the NLRP3 inflammasome, triggering the production and release of pro-inflammatory cytokines such as IL-1β and IL-18 (Calzadilla et al. 2022). Interestingly, Moekotte et al. have previously found an increase in immune cells, such as CD4^+^ T and CD8^+^ T cells and microglia in patients with *CRB1*-related retinopathy (Moekotte et al. 2023). The elevated levels of glycolithocholate and deoxycholate observed in *CRB1* patients may contribute to the inflammatory phenotype described by Moekotte et al., potentially driving increased immune cell activity and the non-infectious uveitis frequently reported in *CRB1* retinopathies (Moekotte et al. 2023). This immune response, which can manifest as vitreous inflammation, cystoid macular oedema, and less frequently, multifocal choroiditis-like lesions, highlights the link between systemic metabolic disturbances and ocular inflammation in these patients (Kiriyama and Nochi 2023, Li et al. 2022, Verhagen et al. 2016). Conversely, the secondary bile acid ursodeoxycholate was significantly lower in *CRB1* patients. Ursodeoxycholic and tauroursodeoxycholic acid (TUDCA) have demonstrated neuroprotective effects in retinitis pigmentosa mice models such as rd10, P23H, and Pde6 (rd1), by inhibiting both caspase-dependent and caspase-independent apoptosis, potentially by preventing the release of apoptosis-inducing factor (AIF) from RPE, photoreceptors, and RGCs (Funabashi et al. 2020, Daruich et al. 2019, Zhang et al.^2012^). TUDCA therapy has shown preservation of photoreceptor number and morphology of inner and outer segments in rd10 mice (Li et al. 2024). Similarly, in a mouse model of Bardet-Biedl syndrome type 1 (RP and obesity), TUDCA not only preserved retinal function and outer nuclear layer thickness but also prevented obesity (Drack et al. 2012). The reduction in these protective bile acids in *CRB1* patients might contribute to a decrease of photoreceptor protection, antioxidant activity, and anti-inflammatory effects in the retina which can lead to a disease progression and potential therapy targets (Daruich et al. 2019).

Recent preclinical and clinical studies suggest that gut microbiome may play a significant role in retinal diseases, including age related macular degeneration (Andriessen et al. 2016), retinopathy of prematurity (Skondra et al. 2020), and aniridia secondary to *PAX6* haploinsufficiency (Cunha 2024). More recently, Peng et al. found that *rd8* mice exhibited weakened adhesion junctions between colonic epithelial cells due to the absence of the Crb1 protein in the intestinal epithelium, leading to impaired gut barrier function (Peng et al. 2024). In *rd8* mice, Crb1 regulate the expression of tight junction proteins such as ZO-1, occludin, and claudin, maintaining the connections between intestinal epithelial cells and providing physical support to the gut barrier (Peng et al. 2024). The authors stated that this barrier dysfunction allowed translocation of gut flora from the lower gastrointestinal tract to the retina, which contributed to bacteria-dependent retinal degeneration (Peng et al. 2024). Comparably, several observations of metabolite dysregulation that might indicate altered gut microbiota composition in *CRB1* patients were noted in this study (i) a significant upregulation in secondary bile acids which are produced by the gut microbiome, and (ii) an upregulation of phenylalanine metabolism which is also modified by the gut microbiome. Dietary phenylalanine was found within normal limits, yet it produces intermediates through the gut microbiome such as phenylpyruvate and 4-hydroxyphenylacetate, both of which were elevated in our study, further supporting the hypothesis of altered gut microbiome-related metabolic activity. However, there was no significant difference in sucrose or butyrate levels between *CRB1* patients and controls. Normally, sucrose is fully broken down and absorbed in the small intestine, with undigested sucrose rarely entering the bloodstream unless gut barrier integrity is compromised (Meddings et al. 1993). The absence of elevated sucrose levels in *CRB1* patients suggests no severe gut barrier disruption in the small intestine (Arnone et al. 2022). Similarly, butyrate, a key short-chain fatty acid absorbed in the colon, showed no significant differences between groups. While this does not directly confirm tight junction integrity, butyrate is known to enhance barrier function by increasing tight junction proteins (claudin-1, ZO-1). Altogether, these findings suggest an absence of strong metabolomic evidence for severe intestinal barrier disruption, rather than definitive normal barrier integrity (Peng et al. 2007).

Overall, bile acid homeostasis and the gut microbiome share an intricate and dynamic relationship, with bile acids influencing microbiome composition and intestinal permeability by regulating inflammation and maintaining tight junction integrity (Calzadilla et al. 2022, Funabashi et al. 2020). They influence occludin expression, activate the Vitamin D receptor to suppress epithelial cell apoptosis and inflammation, and engage TGR5 signalling to regulate tight junctions (Shi et al. 2023). Additionally, bile acids promote enterocyte migration via EGFR- and COX-2-dependent pathways, maintaining gut epithelial integrity (Calzadilla et al. 2022). Although secondary bile acids were significantly higher in *CRB1* patients, this does not necessarily indicate that bile acid dysregulation directly causes gut permeability and bacterial translocation leading to retinal degeneration. Instead, suggests a more complex relationship between *CRB1* and intestinal function. Especially since previous research has shown retinal degeneration regardless of gut involvement. For example, retinal organoids derived from a RP-*CRB1* patient with compound heterozygous mutations c.2843G > A p.(Cys948Tyr) and c.3122T > C p.(Met1041Thr) exhibited OLM disruptions and photoreceptor nuclei protruding above the OLM at DD180 (Quinn, 2019). Similarly, retinal organoids derived from RP-*CRB1* patient homozygous for c.3122T > C p.(Met1041Thr) showed significantly reduced photoreceptor nuclei and ONL thickness compared to isogenic controls (Boon et al. 2023). Comparably, RP-like mouse models (Crb1^KO/C249W^) demonstrated OLM disruptions with photoreceptor nuclei protruding into the subretinal space or into the outer plexiform layer (OPL) leading to the retinal phenotype (Pavert et al. 2007). Additionally, histological analysis of zebrafish models *crb2*^−/−^ done at 48–96 hpf (hours post-fertilisation) showed an absence of retinal layer demarcation and complete cellular disorganisation, with patches of plexiform matter before independent feeding begins (Owen et al. 2023). To date, no other study has shown any signs of bacterial infection aside from *rd8* mice models (Boon et al. 2020, Stehle 2024, Nguyen et al. 2022, Talib et al. 2022, Varela 2023). Further investigation into the gut microbiome and gut permeability in humans with *CRB1* retinopathies is required. Gut dysbiosis can be confirmed by performing microbiome characterisation using 16 S rRNA sequencing; whereas gut permeability can be assessed using methods such as (i) multi-sugar test, which measures sugar excretion in urine to evaluate gut permeability (ii) measurement of intestinal fatty acid-binding protein (I-FABP) which if elevated indicates epithelial damage (iii) citrulline levels, if reduced suggest a loss of enterocyte mass, though dietary influences can complicate their interpretation, and (iv) measurement of serum lipopolysaccharide (LPS), a component of the outer membrane of gram-negative gut bacteria, which if elevated can signal bacterial translocation and compromised barrier integrity (Seethaler et al. 2021, Schoultz and Keita 2020).

Several metabolites associated with energy production appeared to be impacted in the *CRB1* group. Glucose levels were found to be elevated while lactate levels were reduced, despite similar dietary intake of energetics among the *CRB1* patients and controls. Aerobic glycolysis plays a crucial role in energy production in the retinal cells, enhancing metabolism in the retina can make it resistant to underlying genetic insults, which can slow disease progression (Fu et al. 2021, Nelson et al. 2022). In photoreceptors, glucose is processed principally by aerobic glycolysis, from which the lactate byproduct is provided to the RPE and Müller glia for their energetic needs (Fu et al. 2021, Nelson et al. 2022). However, recent findings highlight that lactate is not only a metabolic byproduct; it plays crucial roles in both metabolic and non-metabolic functions. In the retina, lactate serves as the preferred energy source for Müller cells, RGCs, and RPE cells and acts as a ligand to activate GPR81, promoting RGC survival (Fu et al. 2021). Additionally, lactate has now been recognized as an epigenetic modifier, capable of lactylating histone lysine residues, which directly triggers gene transcription from chromatin (Rajala and Rajala 2023). Increased lactate production as a gene-agnostic approach has shown promising results in slowing retinal degeneration in heterozygous P23H rhodopsin knockin mice *Rho*^*P23H/+*^ models by slowing decline of both scotopic and photopic ERG function and ONL thickness seen in OCT (Nelson et al. 2022). Given that *CRB1* gene mutations already result in dysfunction of Müller cells, the additional reduction in lactate levels, an essential energy source for these cells, as seen in this study could further exacerbate disease progression.

Similarly, fatty acid oxidation was found impaired in *CRB1* patients. The levels of free fatty acids were elevated within the *CRB1* group, whereas acylcarnitines were decreased. Fatty acid oxidation plays a role in maintaining photoreceptor metabolic homeostasis (Fu et al. 2021). The reduction of acylcarnitines in *CRB1* patients reflects the inefficient transport of long-chain fatty acids into the mitochondria, leading to impaired β-oxidation and thus affecting effective energy generation (Fu et al. 2021). β-oxidation degrades fatty acids into acetyl-CoA, which enters the Krebs cycle and is oxidized into CO_2_ and water. FADH_2_ and NADH are also released after β-oxidation and Krebs cycle processing which are then used for energy production by the mitochondrial electron transport chain (Fu et al. 2021). In impaired β-oxidation, the ω-oxidation pathway is activated to compensate for the energy shortfall, as further evidenced by the increased levels of dicarboxylic acids in our study. It is noteworthy that there is a direct correlation of lactate and fatty acid oxidation. During the inflammatory stress response, acetyl-CoA generated from fatty acid oxidation can enhance glycolysis through nonenzymatic acetylation, which, in turn, promotes lactate production (Li et al. 2022) which was found reduced on the *CRB1* group. Altogether this showed that there may be defects in fatty acid β-oxidation pathways which may lead to energy deficiency in the retina which can potentially enhance disease progression (Fu et al. 2021).

Antioxidants such as vitamins A, C, E, and carotenoids, can help limit the production of reactive oxygen species (ROS), neutralize free radicals, or enhance enzyme activity to reduce oxidative stress-induced damage to the retina (Ichsan et al. 2022, Kiser and Palczewski 2016). This study showed a significant decrease in α-tocopherol levels in the *CRB1* group compared to controls. This metabolite acts as a potent chain-breaking antioxidant, countering the formation of reactive oxygen species. By inhibiting membrane lipid peroxidation and scavenging lipid peroxyl radicals, it safeguards critical cellular structures from damage caused by oxygen free radicals and lipid peroxidation byproducts (Ichsan et al. 2022). α-tocopherol had been suggested to protect against retinal phototoxicity and central nervous system ischemia (Ichsan et al. 2022). High dose of α-tocopherol has shown positive results in diabetic rat models to prevent diabetes-related vascular damage. Additionally, it has shown in humans to improve retinal vascular hemodynamics (Ichsan et al. 2022). Finally, clinical studies indicate that vitamin E supplements may offer significant benefits to individuals with moderate to severe AMD, while providing only minimal protection during the early stages of the disease (Edwards et al. 2022). Recognising a downregulation of this important pathway and antioxidant could open a possibility of supplementation to delay disease progression in *CRB1* retinopathies.

### Limitations

Due to the rare nature of *CRB1*-related retinopathy, the number of cases was relatively small, which may limit the generalisation of the study results. Ideally, patients at the same stage of the disease should be recruited to more accurately track disease progression. However, the small sample size makes this approach difficult to achieve. This study, intended to provide an exploratory overview of metabolic signatures within a *CRB1* cohort, did not include phenotype stratification. However, future work should be conducted in a phenotype-stratified manner to compare among different phenotypes. A value of q ≤ 0.10 was used given the exploratory nature of this rare-disease cohort, with emphasis placed on consistency of pathway-level effects rather than isolated metabolites. While data were collected over two batches and merged via common QC, batch effects was not assessed. Due to differences in the metabolic status of the population, factors such as age, sex, ethnicity, and lifestyle can influence metabolic levels, making it difficult to control the quality of sample collection, which may affect the identification of differential metabolites. Our conclusions are primarily based on inferences from blood metabolite data, lacking effective validation of the altered pathways. The cross-sectional study design cannot determine the causal relationship between differential metabolites and the disease or its severity, as cross-sectional sampling only captures a snapshot of the metabolic fingerprint at a given point in time. Additionally, non-fasting sampling blood collection and differences in age groups may have contributed to biological and pre-analytical variability. As a result, findings should be interpreted as exploratory. While datasets were harmonised using shared quality-control samples, not all metabolites merged optimally. For example, asparagine, glutamine, and N-acetylvaline could not be analysed in our study to avoid introducing potential technical variability.

## Conclusion

This global metabolomic analysis of patients with *CRB1*-retinopathies revealed systemic metabolic disruptions, particularly in the enterohepatic circulation, antioxidant pathways, and energy metabolism. While *CRB1* mutations are primarily associated with inherited retinal degeneration, our findings suggest broader systemic implications that may contribute to disease progression, inflammation, and severity. Further investigation into gut microbiome composition and intestinal permeability in humans with *CRB1* retinopathies is warranted. These biomarkers could serve as diagnostic tools, potential disease markers, and therapeutic targets. Additionally, identifying the downregulation of key metabolic pathways and antioxidants presents an opportunity for targeted supplementation to potentially slow disease progression in *CRB1*-retinopathies.

## Supplementary Information

Below is the link to the electronic supplementary material.

Supplementary Material 1

Supplementary Material 2

Supplementary Material 3

## Data Availability

Data is provided within the manuscript or supplementary information files.
