# Supplementary material for: Metabolomic signatures suggest altered bile acid and energy metabolism in CRB1- retinopathies
Source: Metabolomics. 2026 Apr 17;22(3):52. doi: 10.1007/s11306-026-02415-7 (PMC13090204; doi:10.1007/s11306-026-02415-7)
Supplement: Supplementary file 1 — Supplementary Material 1 [file 11306_2026_2415_MOESM1_ESM.pdf]

Supplementary Materials 1

MATERIALS AND METHODS

HUMAN PLASMA METABOLOMICS ANALYSIS

Each sample was accessioned into the Metabolon Laboratory Information. Management System (LIMS) and assigned a unique identifier associated with the original de-identified study code number. The identifier was used to track all sample handling, tasks and results. Samples were prepared using the automated MicroLab STAR® system from Hamilton Company (Reno, NV, USA). Several recovery standards were added prior to the first step in the extraction process for quality control purposes. To remove protein, dissociate small molecules bound to protein or trapped in the precipitated protein matrix, and to recover chemically diverse metabolites, proteins were precipitated with methanol under vigorous shaking for 2 min (Glen 15 Mills GenoGrinder 2000) followed by centrifugation. The resulting extract was divided into five fractions: two for analysis by two separate reverse phase (RP)/UPLC-MS/MS methods with positive ion mode electrospray ionization (ESI), one for analysis by RP/UPLC-MS/MS with negative ion mode ESI, one for analysis by HILIC/UPLC-MS/MS with negative ion mode ESI, and one sample was reserved for backup. Samples were placed briefly on a TurboVap® (Zymark) to remove the organic solvent. The sample extracts were stored overnight under nitrogen before preparation for UPLC-MS/MS analysis. The UPLC-MS/MS platform utilised a Waters ACQUITY ultra-performance liquid chromatography (UPLC) and a Thermo Scientific Q-Exactive high resolution/accurate mass spectrometer interfaced with a heated electrospray ionization (HESI-II) source and Orbitrap mass analyzer operated at 35,000 mass resolution. The dried sample extract was reconstituted in acidic or basic UPLC- compatible solvents, each of which contained 11 to 13 injection standards at fixed concentrations (14). Two aliquots were analysed using acidic positive ion conditions, chromatographically optimised for hydrophilic or hydrophobic compounds and the other using basic negative ion optimised conditions, using separate dedicated columns (Waters UPLC BEH C18-2.1x100 mm, 1.7 µm). Extracts reconstituted in acidic conditions were gradient eluted using water and methanol, containing 0.05% perfluoropentanoic acid (PFPA) and 0.1% formic acid (FA) for hydrophilic compounds or water, methanol, acetonitrile, 0.05% 36 PFPA and 0.01% FA for hydrophobic compounds. Basic extracts were also gradient eluted using methanol and water, but with the addition of 6.5mM Ammonium Bicarbonate at pH 8. The fourth aliquot was analysed via negative ionization following elution from a HILIC column (Waters UPLC BEH Amide 40 2.1x150 mm, 1.7 µm) using a gradient consisting of water and acetonitrile with 10 mM Ammonium Formate, pH 10.8. The MS analysis alternated between MS and data-dependent MS/MS scans using

dynamic exclusion and the scan range covered 70-1000 m/z. Raw data was extracted and peak-identified as per Metabolon's hardware and software. Biochemical identifications were based on three criteria: retention index within a narrow RI window of the proposed identification, accurate mass match to the library +/- 10 ppm, and the MS/MS forward and reverse scores between 3 the experimental data and authentic standards. The MS/MS scores were based on a comparison of the ions present in the experimental spectrum to the ions present in the library spectrum. More than 3300 commercially available purified standard compounds have been acquired and registered into LIMS for analysis on all platforms for determination of their analytical characteristics (REF).

Supplementary Table 1. Summary of subject demographics, genetic results, and clinical characteristics of the 25 patients with biallelic pathogenic variants in *CRB1*.

Family number	Subject	Gender	Ethnicity	Age	Phenotype	Zygosity	Variant 1 cDNA<br>Variant 1 protein	Variant 2 cDNA<br>Variant 2 protein
45590	01	F	Black	29	MD	Homozygous	c.2506C>A p.Pro836Thr	
46120	02	F	White	17	EOSRD/LCA	Heterozygous	c.455G>A<br>p.Cys152Tyr	c.3014A>T<br>p.Asp1005Val
35083	03	M	White	39	MD	Heterozygous	c.498_506del<br>p.Ile167_Gly169del	c.4142C>G<br>p.Pro1381Arg
43560	04	F	White	48	MD	Heterozygous	c.498_506del<br>p.Ile167_Gly169del	c.1696G>T<br>p.Glu556Ter
38236	05	M	White	47	MD	Heterozygous	c.498_506del<br>p.Ile167_Gly169del	c.584G>T<br>p.Cys195Phe
16285	06	M	White	26	EOSRD/LCA	Homozygous	c.2843G>A p.Cys948Tyr	
Z889804	07	M	Asian	13	CORD	Heterozygous	c.498_506del<br>p.Ile167_Gly169del	c.4005+1G>A<br>N/A
32309	08	M	White	15	MD	Heterozygous	c.498_506del<br>p.Ile167_Gly169del	c.1576C>T<br>p.Arg525*
42270	09	M	White	52	MD	Heterozygous	c.498_506del<br>p.Ile167_Gly169del	c.2401A>T<br>p.Lys801*
31953	10	F	White	16	EOSRD/LCA	Heterozygous	c.2548G>A<br>p.Gly850Ser	c.4006-10A>G<br>N/A
37161	11	M	White	17	MD	Heterozygous	c.498_506del<br>p.Ile167_Gly169del	c.2308G>T<br>p.Gly770Cys
46830	12	M	White	10	EOSRD/LCA	Heterozygous	c.2843G>A<br>p.Cys948Tyr	c.1712A>C<br>p.Glu571Ala
44092	13	F	White	11	MD	Heterozygous	c.498_506del<br>p.Ile167_Gly169del	c.2843G>A<br>p.Cys948Tyr
35283	14	M	White	11	EOSRD/LCA	Homozygous	c.2843G>A p.Cys948Tyr	
29882	15	F	White	16	EOSRD/LCA	Homozygous	c.14559T>C p.Ser487Pro	
32038	16	M	White	40	CORD	Heterozygous	c.498_506del<br>p.Ile167_Gly169del	c.1431delG<br>p.Ser478Profs*24
33707	17	M	White	29	MD	Heterozygous	c.498_506del<br>p.Ile167_Gly169del	c.3827_3828del<br>p.Glu1276Valfs*4
47941	18	M	White	18	EOSRD/LCA	Heterozygous	c.2291G>A p.Arg764His	

21819	19	M	White	33	CORD	Homozygous	c.470G>C p.C157Sp.Cys157Ser	c.2506C>A p.Pro836Thr
35229	20	F	White	34	MD	Heterozygous	c.498_506del p.Ile167_Gly169del	c.2290C>T p.Arg764Cys
27040	21	F	White	5	EOSRD/LCA	Heterozygous	c.2401A>T p.Lys801*	c.2688T>A p.Cys896*
28183	22	F	White	5	EOSRD/LCA	Heterozygous	c.1339dupC p.His447Prof*7	c.2401A>T p.Lys801*
	23	F	White	41	MD	Heterozygous	c.498_506del p.Ile167_Gly169del	c.3718T>A p.Cys1240Ser
18067	24	M	White	16	EOSRD/LCA	Heterozygous	c.2401A>T p.Lys801*	c.2872delAG p.S958fs
24729	25	M	Asian	13	EOSRD/LCA	Heterozygous	c.2512A>T p.Lys838*	c.3676G>T p.Gly1226*

Supplementary Table 2. Dietary intake of *CRB1* patients and controls.

**Food/nutrient**	***CRB1* patients Mean (±SD)**	**Controls Mean (±SD)**	***p*-value**
Fruits (portions/day)	2.12 (1.80)	2.17 (1.42)	0.65
Vegetables (portions/day)	5.03 (3.78)	4.52 (2.77)	0.64
Leafy vegetables (portions/day)	1.03 (0.91)	0.94 (0.55)	0.89
Meat (portions/day)	1.30 (0.87)	1.04 (0.53)	0.36
Fish (portions/day)	0.43 (0.35)	0.37 (0.28)	0.39
Milk (pints/day)	0.31 (0.36)	0.44 (0.49)	0.41
Other dairy (portions/day)	1.16 (0.80)	0.95 (0.94)	0.07
Cereal foods (portions/day)	5.84 (5.02)	4.66 (2.66)	0.39
Carbohydrate (g)	306.9 (207.7)	252.6 (97.1)	0.48
Carbohydrate as a % of total energy	48.57 (6.87)	47.25 (5.79)	0.59
Total sugars (g)	124.1 (78.8)	111.3 (47.9)	0.73
Glucose (g)	24.0 (16.7)	21.8 (9.2)	0.81
Fructose (g)	26.6 (18.3)	24.5 (11.4)	0.91
Sucrose (g)	52.4 (35.8)	42.5 (23.0)	0.16
Maltose (g)	4.89 (2.99)	4.00 (2.19)	0.17
Lactose (g)	15.0 (12.7)	17.2 (14.7)	0.75
Starch (g)	180.7 (143.0)	139.7 (61.5)	0.41
Energy (kJ)	9948 (5938)	8576 (3268)	0.37
Total fat as a % of total energy	35.0 (5.3)	34.6 (4.6)	0.49
Saturated fat as a % of total energy	12.8 (2.5)	12.6 (2.5)	0.61
Monounsaturated fat as a % of total energy	12.5 (2.5)	12.1 (1.7)	0.54
PUFAs as a % of total energy	6.77 (2.46)	6.95 (2.04)	0.53
N3 PUFAs as a % of total energy	0.72 (0.22)	0.73 (0.22)	0.88
Protein as a % of total energy	16.8 (3.4)	17.3 (2.9)	0.29
Fibre (g)	21.7 (16.7)	18.0 (7.1)	0.77

Alcohol (g)	4.69 (10.17)	7.67 (14.00)	0.24
Vitamin C (mg)	170.4 (132.6)	140.5 (80.0)	0.39
Thiamine (mg)	1.99 (1.31)	1.64 (0.62)	0.33
Riboflavin (mg)	2.18 (1.57)	2.06 (0.94)	0.69
Niacin (mg)	24.5 (14.6)	21.8 (8.1)	0.94
Vitamin B6 (mg)	2.18 (1.34)	1.89 (0.72)	0.35
Vitamin B12 (µg)	7.29 (6.82)	6.61 (3.68)	0.74
Folate (µg)	355 (256)	296 (125)	0.42
Carotene (µg)	3839 (3164)	3969 (2312)	0.47
Caffeine (mg)	75.0 (75.3)	174.8 (134.2)	<0.01*

*Mann-Whitney test
