# Supplementary material for: Metabolomic signatures suggest altered bile acid and energy metabolism in CRB1- retinopathies
Source: Metabolomics. 2026 Apr 17;22(3):52. doi: 10.1007/s11306-026-02415-7 (PMC13090204; doi:10.1007/s11306-026-02415-7)
Supplement: Supplementary file 3 — Supplementary Material 3 [file 11306_2026_2415_MOESM3_ESM.pdf]

Study Number: | | |

# YOUR DIET LAST YEAR

For each food there is an amount shown, either a "medium serving" or an example of a serving such as "a slice" or "a teaspoon". Please put a cross (×) in the box to indicate how often, **on average**, you have eaten a specified amount of food over the last 12 months. If you complete a box <u>incorrectly</u> please fill the wrong one. e.g. ■ and put a cross in the correct box.

The rest of this page shows examples of how to fill in the questionnaire.

**These questions are all about the food you have eaten over the last year.**

**EXAMPLE 1**

For white bread the serving size is one slice or roll, so if you ate 2 to 4 slices a week on average, over the last 12 months, you should put a cross in the column headed "2-4 per week".

| FOODS AND AMOUNTS | Never or less than once/month | 1-3 per month | Once a week | 2-4 per week | 5-6 per week | Once a day | 2-3 per day | 4-5 per day | 6+ per day |
|---|---|---|---|---|---|---|---|---|---|
| **BREAD** (one slice or roll) | | | | | | | | | |
| White bread and rolls | ☐ | ☐ | ☐ | × | ☐ | ☐ | ☐ | ☐ | ■ example of mistake |

AVERAGE USE IN THE LAST TWELVE MONTHS

**EXAMPLE 2**

For chips, the serving size is a small side plate so if you ate a serving of chips twice a week, over the last 12 months, you should put a cross in the column headed "2-4 per week".

| FOODS AND AMOUNTS | Never or less than once/month | 1-3 per month | Once a week | 2-4 per week | 5-6 per week | Once a day | 2-3 per day | 4-5 per day | 6+ per day |
|---|---|---|---|---|---|---|---|---|---|
| **POTATOES** (one small side plate) | | | | | | | | | |
| Chips | ☐ | ☐ | ☐ | × | ☐ | ☐ | ☐ | ☐ | ☐ |

AVERAGE USE IN THE LAST TWELVE MONTHS

**EXAMPLE 3**

For very seasonal fruits such as strawberries and raspberries you should estimate your average intake in summer, so if you ate strawberries or raspberries about once a week in summer you should put a cross in the column headed "once a week".

| FOODS AND AMOUNTS | Never or less than once/month | 1-3 per month | Once a week | 2-4 per week | 5-6 per week | Once a day | 2-3 per day | 4-5 per day | 6+ per day |
|---|---|---|---|---|---|---|---|---|---|
| **FRUIT** (1 fruit or handful) | | | | | | | | | |
| Strawberries, raspberries, kiwi fruit | ☐ | ☐ | × | ☐ | ☐ | ☐ | ☐ | ☐ | ☐ |

AVERAGE USE IN THE LAST TWELVE MONTHS

EXAMPLE

Study Number: ☐☐☐

## START OF QUESTIONNAIRE

1a. On average do you think you eat the same, more or less than someone of your own age and sex? ☐ Same ☐ More ☐ Less
**Please put a cross (×) in only one box.**

1b. On an average day how many portions of fruit do you eat? ☐

1c. On an average day how many portions of vegetables do you eat? ☐

Please estimate your average food use as best you can, and please answer every question - do not leave ANY lines blank. **PLEASE PUT A CROSS (×) ON EVERY LINE**

**FOODS AND AMOUNTS** **AVERAGE USE IN THE LAST TWELVE MONTHS**

| DAIRY PRODUCTS AND FATS | Never or less than once/month | 1-3 per month | Once a week | 2-4 per week | 5-6 per week | Once a day | 2-3 per day | 4-5 per day | 6+ per day |
|---|---|---|---|---|---|---|---|---|---|
| Single or sour cream (tablespoon) | ☐ | ☐ | ☐ | ☐ | ☐ | ☐ | ☐ | ☐ | ☐ |
| Double or clotted cream (tablespoon) | ☐ | ☐ | ☐ | ☐ | ☐ | ☐ | ☐ | ☐ | ☐ |
| Low fat yoghurt, fromage frais (small pot) | ☐ | ☐ | ☐ | ☐ | ☐ | ☐ | ☐ | ☐ | ☐ |
| Full fat or Greek yoghurt (small pot) | ☐ | ☐ | ☐ | ☐ | ☐ | ☐ | ☐ | ☐ | ☐ |
| Dairy desserts (small pot) e.g. chocolate mousse, cream caramels | ☐ | ☐ | ☐ | ☐ | ☐ | ☐ | ☐ | ☐ | ☐ |
| Cheese, e.g. cheddar, brie, edam (matchbox size) | ☐ | ☐ | ☐ | ☐ | ☐ | ☐ | ☐ | ☐ | ☐ |
| Low fat cheese e.g. reduced fat cheddar (matchbox size) | ☐ | ☐ | ☐ | ☐ | ☐ | ☐ | ☐ | ☐ | ☐ |
| Cottage cheese, low fat soft cheese (2 tablespoons) | ☐ | ☐ | ☐ | ☐ | ☐ | ☐ | ☐ | ☐ | ☐ |
| Eggs as boiled, fried, scrambled, etc. (one) | ☐ | ☐ | ☐ | ☐ | ☐ | ☐ | ☐ | ☐ | ☐ |
| Quiche (slice) | ☐ | ☐ | ☐ | ☐ | ☐ | ☐ | ☐ | ☐ | ☐ |
| Low calorie, low fat salad cream (tablespoon) | ☐ | ☐ | ☐ | ☐ | ☐ | ☐ | ☐ | ☐ | ☐ |
| Full fat salad cream, mayonnaise (tablespoon) | ☐ | ☐ | ☐ | ☐ | ☐ | ☐ | ☐ | ☐ | ☐ |
| French dressing (tablespoon) | ☐ | ☐ | ☐ | ☐ | ☐ | ☐ | ☐ | ☐ | ☐ |
| Other salad dressing (tablespoon) | ☐ | ☐ | ☐ | ☐ | ☐ | ☐ | ☐ | ☐ | ☐ |

Study Number:

**FOODS AND AMOUNTS** **AVERAGE USE IN THE LAST TWELVE MONTHS**

| The following list refers to **DAIRY PRODUCTS AND FATS** that you put on bread or cooked vegetables | Never or less than once/month | 1-3 per month | Once a week | 2-4 per week | 5-6 per week | Once a day | 2-3 per day | 4-5 per day | 6+ per day |
|---|---|---|---|---|---|---|---|---|---|
| Butter (teaspoon) | ☐ | ☐ | ☐ | ☐ | ☐ | ☐ | ☐ | ☐ | ☐ |
| Reduced fat butter (teaspoon) | ☐ | ☐ | ☐ | ☐ | ☐ | ☐ | ☐ | ☐ | ☐ |
| Block margarine, e.g. Stork, Krona (teaspoon) | ☐ | ☐ | ☐ | ☐ | ☐ | ☐ | ☐ | ☐ | ☐ |
| Polyunsaturated margarine, e.g. Flora, sunflower (teaspoon) | ☐ | ☐ | ☐ | ☐ | ☐ | ☐ | ☐ | ☐ | ☐ |
| Olive oil spread (teaspoon) | ☐ | ☐ | ☐ | ☐ | ☐ | ☐ | ☐ | ☐ | ☐ |
| Other soft margarine, dairy spreads, e.g. Blue Band, Clover (teaspoon) | ☐ | ☐ | ☐ | ☐ | ☐ | ☐ | ☐ | ☐ | ☐ |
| Low fat spread, e.g. Outline, Gold (teaspoon) | ☐ | ☐ | ☐ | ☐ | ☐ | ☐ | ☐ | ☐ | ☐ |
| Very low fat spread (teaspoon) e.g. Diet Flora | ☐ | ☐ | ☐ | ☐ | ☐ | ☐ | ☐ | ☐ | ☐ |
| Cholesterol lowering fat spreads e.g. Benecol (teaspoon) | ☐ | ☐ | ☐ | ☐ | ☐ | ☐ | ☐ | ☐ | ☐ |

**FRUIT - For seasonal fruits marked*, please estimate your average use in summer**

| | | | | | | | | | |
|---|---|---|---|---|---|---|---|---|---|
| Apples (1 fruit) | ☐ | ☐ | ☐ | ☐ | ☐ | ☐ | ☐ | ☐ | ☐ |
| Pears (1 fruit) | ☐ | ☐ | ☐ | ☐ | ☐ | ☐ | ☐ | ☐ | ☐ |
| Oranges, satsumas, mandarins (1 fruit) | ☐ | ☐ | ☐ | ☐ | ☐ | ☐ | ☐ | ☐ | ☐ |
| Grapefruit (half) | ☐ | ☐ | ☐ | ☐ | ☐ | ☐ | ☐ | ☐ | ☐ |
| Bananas (1fruit) | ☐ | ☐ | ☐ | ☐ | ☐ | ☐ | ☐ | ☐ | ☐ |
| Grapes (handful) | ☐ | ☐ | ☐ | ☐ | ☐ | ☐ | ☐ | ☐ | ☐ |
| Melon (1 slice) | ☐ | ☐ | ☐ | ☐ | ☐ | ☐ | ☐ | ☐ | ☐ |
| * Peaches, plums, apricots (1 fruit) | ☐ | ☐ | ☐ | ☐ | ☐ | ☐ | ☐ | ☐ | ☐ |
| * Strawberries, raspberries, other berries, kiwi fruit (one fruit or handful) | ☐ | ☐ | ☐ | ☐ | ☐ | ☐ | ☐ | ☐ | ☐ |
| Tinned fruit (handful) | ☐ | ☐ | ☐ | ☐ | ☐ | ☐ | ☐ | ☐ | ☐ |
| Dried fruit, e.g. raisins, prunes (heaped tablespoon) | ☐ | ☐ | ☐ | ☐ | ☐ | ☐ | ☐ | ☐ | ☐ |

Please check that you have a cross (×) on EVERY line

Study Number:

## FOODS AND AMOUNTS

## AVERAGE USE IN THE LAST TWELVE MONTHS

### VEGETABLES

Fresh, frozen or tinned (handful)

| | Never or less than once/month | 1-3 per month | Once a week | 2-4 per week | 5-6 per week | Once a day | 2-3 per day | 4-5 per day | 6+ per day |
|---|---|---|---|---|---|---|---|---|---|
| Carrots | | | | | | | | | |
| Spinach | | | | | | | | | |
| Broccoli, spring green, kale | | | | | | | | | |
| Brussel sprouts | | | | | | | | | |
| Cabbage | | | | | | | | | |
| Peas | | | | | | | | | |
| Green beans, broad beans, runner beans | | | | | | | | | |
| Baked beans | | | | | | | | | |
| Marrow, courgettes | | | | | | | | | |
| Cauliflower | | | | | | | | | |
| Parsnips, turnips, swedes | | | | | | | | | |
| Leeks | | | | | | | | | |
| Onions | | | | | | | | | |
| Garlic (clove) | | | | | | | | | |
| Mushrooms | | | | | | | | | |
| Sweet peppers | | | | | | | | | |
| Beansprouts | | | | | | | | | |
| Green salad, lettuce, cucumber, celery | | | | | | | | | |
| Watercress | | | | | | | | | |
| Tomatoes | | | | | | | | | |
| Sweetcorn | | | | | | | | | |
| Beetroot | | | | | | | | | |
| Coleslaw | | | | | | | | | |
| Avocado | | | | | | | | | |
| Pulses e.g. lentils, beans, peas | | | | | | | | | |
| Meat substitutes e.g. tofu, soyameat, textured vegetable protein, vegeburger | | | | | | | | | |

Please check that you have a cross (×) on EVERY line

Study Number:

**FOODS AND AMOUNTS** | **AVERAGE USE IN THE LAST TWELVE MONTHS**

| **MEAT AND FISH** (half a small side plate) | Never or less than once/month | 1-3 per month | Once a week | 2-4 per week | 5-6 per week | Once a day | 2-3 per day | 4-5 per day | 6+ per day |
|---|---|---|---|---|---|---|---|---|---|
| Beef: roast, steak, mince, stew or casserole | ☐ | ☐ | ☐ | ☐ | ☐ | ☐ | ☐ | ☐ | ☐ |
| Beefburgers | ☐ | ☐ | ☐ | ☐ | ☐ | ☐ | ☐ | ☐ | ☐ |
| Pork: roast, chops or stew | ☐ | ☐ | ☐ | ☐ | ☐ | ☐ | ☐ | ☐ | ☐ |
| Lamb: roast, chops or stew | ☐ | ☐ | ☐ | ☐ | ☐ | ☐ | ☐ | ☐ | ☐ |
| Chicken or other poultry e.g. turkey | ☐ | ☐ | ☐ | ☐ | ☐ | ☐ | ☐ | ☐ | ☐ |
| Bacon or gammon | ☐ | ☐ | ☐ | ☐ | ☐ | ☐ | ☐ | ☐ | ☐ |
| Ham, cured meats & chorizo | ☐ | ☐ | ☐ | ☐ | ☐ | ☐ | ☐ | ☐ | ☐ |
| Corned Beef, Spam, luncheon meats | ☐ | ☐ | ☐ | ☐ | ☐ | ☐ | ☐ | ☐ | ☐ |
| Sausages | ☐ | ☐ | ☐ | ☐ | ☐ | ☐ | ☐ | ☐ | ☐ |
| Savoury pies, e.g. meat pie, pork pie, pasties, steak & kidney pie, sausage rolls | ☐ | ☐ | ☐ | ☐ | ☐ | ☐ | ☐ | ☐ | ☐ |
| Liver, liver pate, liver sausage | ☐ | ☐ | ☐ | ☐ | ☐ | ☐ | ☐ | ☐ | ☐ |
| Fried fish in batter, as in fish and chips | ☐ | ☐ | ☐ | ☐ | ☐ | ☐ | ☐ | ☐ | ☐ |
| Fish fingers, fish cakes & breaded fish | ☐ | ☐ | ☐ | ☐ | ☐ | ☐ | ☐ | ☐ | ☐ |
| Other white fish, fresh or frozen, e.g. cod, plaice, sole, haddock, halibut | ☐ | ☐ | ☐ | ☐ | ☐ | ☐ | ☐ | ☐ | ☐ |
| Oily fish, fresh or canned, e.g. tuna, mackerel, kippers, salmon, sardines, herring | ☐ | ☐ | ☐ | ☐ | ☐ | ☐ | ☐ | ☐ | ☐ |
| Shellfish, e.g. crab, prawns, mussels | ☐ | ☐ | ☐ | ☐ | ☐ | ☐ | ☐ | ☐ | ☐ |
| Fish roe, taramasalata | ☐ | ☐ | ☐ | ☐ | ☐ | ☐ | ☐ | ☐ | ☐ |

Please check that you have a cross (×) on EVERY line

Study Number:

**FOODS AND AMOUNTS** **AVERAGE USE IN THE LAST TWELVE MONTHS**

| **BREAD AND SAVOURY BISCUITS** (one slice, or roll, or biscuit) | Never or less than once/month | 1-3 per month | Once a week | 2-4 per week | 5-6 per week | Once a day | 2-3 per day | 4-5 per day | 6+ per day |
|---|---|---|---|---|---|---|---|---|---|
| White bread/rolls | | | | | | | | | |
| Brown bread/rolls | | | | | | | | | |
| Wholemeal & granary bread/rolls | | | | | | | | | |
| Cream crackers, savoury biscuits | | | | | | | | | |
| Crispbread, e.g. Ryvita | | | | | | | | | |
| Naan, poppadoms, flour tortillas | | | | | | | | | |

| **SOUPS, SAUCES AND SPREADS** | | | | | | | | | |
|---|---|---|---|---|---|---|---|---|---|
| Vegetable soups (bowl) | | | | | | | | | |
| Meat soups (bow ) (to nc ude meat and vegetable soups) | | | | | | | | | |
| Sauces, e.g. wh te sauce, cheese sauce, gravy (tablespoon) | | | | | | | | | |
| Tomato ketchup (tablespoon) | | | | | | | | | |
| P ck es, chutney (tab espoon) | | | | | | | | | |
| Marmite, Bovril (teaspoon) | | | | | | | | | |
| Jam, marmalade, honey (teaspoon) | | | | | | | | | |
| Peanut butter (teaspoon) | | | | | | | | | |

Please check that you have a cross (×) on EVERY line

Study Number:

**FOODS AND AMOUNTS** **AVERAGE USE IN THE LAST TWELVE MONTHS**

| **SWEETS AND SNACKS** (medium serving) | Never or less than once/month | 1-3 per month | Once a week | 2-4 per week | 5-6 per week | Once a day | 2-3 per day | 4-5 per day | 6+ per day |
|---|---|---|---|---|---|---|---|---|---|
| Cakes e.g. fruit, sponge, home baked | | | | | | | | | |
| Cakes e.g. fruit, sponge, ready made | | | | | | | | | |
| Buns, pastries e.g. scones, flapjacks, croissants, doughnuts, home baked | | | | | | | | | |
| Buns, pastries e.g. scones, flapjacks, croissants, doughnuts, ready made | | | | | | | | | |
| Fruit pies, tarts, crumbles, home baked | | | | | | | | | |
| Fruit pies, tarts, crumbles, ready made | | | | | | | | | |
| Sponge puddings, home baked | | | | | | | | | |
| Sponge puddings, ready made | | | | | | | | | |
| Milk puddings e.g. rice, custard, trifle | | | | | | | | | |
| Ice cream, choc ices | | | | | | | | | |
| Sweet biscuits, chocolate, e.g. digestive (one) | | | | | | | | | |
| Sweet biscuits, plain, e.g. Nice, ginger (one) | | | | | | | | | |
| Reduced fat biscuits e.g. Go Ahead, Highlights (one small packet or one small bar/biscuit) | | | | | | | | | |
| Cereal bars (one) | | | | | | | | | |
| White or milk chocolates, single or squares (one) | | | | | | | | | |
| Dark chocolates, single or squares (one) | | | | | | | | | |
| Chocolate snack bars e.g. Mars, Crunchie (one) | | | | | | | | | |
| Sweets, toffees, mints (small packet) | | | | | | | | | |
| Sugar added to tea, coffee, cereal (teaspoon) | | | | | | | | | |

Please check that you have a cross (×) on EVERY line

Study Number:

**FOODS AND AMOUNTS** **AVERAGE USE IN THE LAST TWELVE MONTHS**

| SWEETS AND SNACKS | Never or less than once/month | 1-3 per month | Once a week | 2-4 per week | 5-6 per week | Once a day | 2-3 per day | 4-5 per day | 6+ per day |
|---|---|---|---|---|---|---|---|---|---|
| Crisps or other packet snacks, e.g. Wotsits (one packet) | | | | | | | | | |
| Salted nuts e.g. peanuts, cashews (handful) | | | | | | | | | |
| Unsalted nuts, e.g. brazil, walnuts (handful) | | | | | | | | | |
| Seeds e.g. Sunflower, pumpkin (tablespoon) | | | | | | | | | |

**CEREAL** (one bowl)

| | | | | | | | | | |
|---|---|---|---|---|---|---|---|---|---|
| Porridge, Readybreak, oats | | | | | | | | | |
| Breakfast cereal e.g. Cornflakes, Rice Krispies | | | | | | | | | |
| Sugar topped cereals e.g. Frosties | | | | | | | | | |
| Muesli | | | | | | | | | |
| High Fibre cereals e.g. Branflakes, All Bran, Fruit and Fibre | | | | | | | | | |

**POTATOES, RICE AND PASTA**
(one small side plate)

| | | | | | | | | | |
|---|---|---|---|---|---|---|---|---|---|
| Boiled, mashed, instant or one jacket potato | | | | | | | | | |
| Chips, roast potatoes | | | | | | | | | |
| Potato salad | | | | | | | | | |
| White rice | | | | | | | | | |
| Brown rice | | | | | | | | | |
| White or green pasta, e.g. spaghetti, macaroni, noodles | | | | | | | | | |
| Wholemeal pasta | | | | | | | | | |
| Lasagne, moussaka | | | | | | | | | |
| Pizza (one slice) | | | | | | | | | |

**Please check that you have a cross (×) on EVERY line**

Study Number:

## FOODS AND AMOUNTS

## AVERAGE USE IN THE LAST TWELVE MONTHS

| DRINKS | Never or less than once/month | 1-3 per month | Once a week | 2-4 per week | 5-6 per week | Once a day | 2-3 per day | 4-5 per day | 6+ per day |
|---|---|---|---|---|---|---|---|---|---|
| Tea (cup) | ☐ | ☐ | ☐ | ☐ | ☐ | ☐ | ☐ | ☐ | ☐ |
| Green tea (cup) | ☐ | ☐ | ☐ | ☐ | ☐ | ☐ | ☐ | ☐ | ☐ |
| Fruit tea (cup) | ☐ | ☐ | ☐ | ☐ | ☐ | ☐ | ☐ | ☐ | ☐ |
| Coffee, instant or ground (cup) | ☐ | ☐ | ☐ | ☐ | ☐ | ☐ | ☐ | ☐ | ☐ |
| Coffee, decaffeinated (cup) | ☐ | ☐ | ☐ | ☐ | ☐ | ☐ | ☐ | ☐ | ☐ |
| Coffee whitener, e.g. Coffee-mate (teaspoon) | ☐ | ☐ | ☐ | ☐ | ☐ | ☐ | ☐ | ☐ | ☐ |
| Cocoa, hot chocolate (cup) | ☐ | ☐ | ☐ | ☐ | ☐ | ☐ | ☐ | ☐ | ☐ |
| Low fat hot chocolate (cup) | ☐ | ☐ | ☐ | ☐ | ☐ | ☐ | ☐ | ☐ | ☐ |
| Horlicks, Ovaltine (cup) | ☐ | ☐ | ☐ | ☐ | ☐ | ☐ | ☐ | ☐ | ☐ |
| White wine (small glass) | ☐ | ☐ | ☐ | ☐ | ☐ | ☐ | ☐ | ☐ | ☐ |
| Red wine (small glass) | ☐ | ☐ | ☐ | ☐ | ☐ | ☐ | ☐ | ☐ | ☐ |
| Beer, lager or cider (half pint) | ☐ | ☐ | ☐ | ☐ | ☐ | ☐ | ☐ | ☐ | ☐ |
| Port, sherry, vermouth, liqueurs (pub measure) | ☐ | ☐ | ☐ | ☐ | ☐ | ☐ | ☐ | ☐ | ☐ |
| Spirits, e.g. gin, brandy, whisky, vodka (pub measure) | ☐ | ☐ | ☐ | ☐ | ☐ | ☐ | ☐ | ☐ | ☐ |
| Low calorie or diet fizzy soft drinks (cup) | ☐ | ☐ | ☐ | ☐ | ☐ | ☐ | ☐ | ☐ | ☐ |
| Fizzy soft drinks, e.g. Coca Cola, lemonade (cup) | ☐ | ☐ | ☐ | ☐ | ☐ | ☐ | ☐ | ☐ | ☐ |
| Pure fruit juice (100%) e.g. orange, apple juice (cup) | ☐ | ☐ | ☐ | ☐ | ☐ | ☐ | ☐ | ☐ | ☐ |
| Fruit squash or cordial (cup) | ☐ | ☐ | ☐ | ☐ | ☐ | ☐ | ☐ | ☐ | ☐ |
| Smoothies (cup) | ☐ | ☐ | ☐ | ☐ | ☐ | ☐ | ☐ | ☐ | ☐ |

Study Number: ☐☐☐

## YOUR DIET LAST YEAR, continued

2. Are there any **OTHER** foods which you ate more than once a week over the last year?

No ☐ → **GO TO** question 3 Yes ☐ **If yes**, please list below

| Food | Usual serving size | Number of times eaten each week |
|---|---|---|
| | | |
| | | |
| | | |
| | | |

3. Have you drunk milk or a milk substitute over the past year? (including in hot drinks)

No ☐ → **GO TO** question 6 Yes ☐ → **GO TO** question 4

4. What type of milk or milk substitute did you most often use over the last year?

**Select one only**

| | | | |
|---|---|---|---|
| Full cream | ☐ | Semi- skimmed | ☐ |
| Skimmed | ☐ | Channel Islands | ☐ |
| Dried milk | ☐ | Soya | ☐ |

Other (please specify) ____________

5. About how much milk did you drink each day, including milk with tea, coffee, cereals etc?

| | | | |
|---|---|---|---|
| Less than a quarter of a pint | ☐ | One Pint | ☐ |
| Half a pint | ☐ | More than one pint | ☐ |
| Three quarters of a pint | ☐ | | |

6. Have you taken any vitamins, minerals, fish oils, fibre or other food supplements during the past year?

No ☐ You have finished the questionnaire. Many thanks! Yes ☐ → **GO TO** question 7

Study Number:

7. Please list any vitamins, minerals, fish oils, fibre or other food supplements taken during the past year. If you have taken more than 6 types of supplement please list the 6 most frequently consumed brands.

**Vitamin supplements**

**Average frequency**
Cross **one** box per line to show how often on average you consumed supplements

| **Name and brand** Please list full name and **brand** | **Number** Please state number of pills, capsules or teaspoons consumed | Never or less than once/month | 1-3 per month | Once a week | 2-4 per week | 5-6 per week | Once a day | 2-3 per day | 4-5 per day | 6+ per day |
|---|---|---|---|---|---|---|---|---|---|---|
| | 1 - 9 | ☐ | ☐ | ☐ | ☐ | ☐ | ☐ | ☐ | ☐ | ☐ |
| | 1 - 9 | ☐ | ☐ | ☐ | ☐ | ☐ | ☐ | ☐ | ☐ | ☐ |
| | 1 - 9 | ☐ | ☐ | ☐ | ☐ | ☐ | ☐ | ☐ | ☐ | ☐ |
| | 1 - 9 | ☐ | ☐ | ☐ | ☐ | ☐ | ☐ | ☐ | ☐ | ☐ |
| | 1 - 9 | ☐ | ☐ | ☐ | ☐ | ☐ | ☐ | ☐ | ☐ | ☐ |
| | 1 - 9 | ☐ | ☐ | ☐ | ☐ | ☐ | ☐ | ☐ | ☐ | ☐ |

***Many thanks for completing this questionnaire.***

***Please return the completed questionnaire, using the pre-paid envelope provided.***
